# Leveraging multi-scale feature integration in UNet and FPN for semantic segmentation of lung nodules

**DOI:** 10.3389/frai.2025.1682171

**Published:** 2025-10-30

**Authors:** Sarah Prithvika, Jani Anbarasi, Modigari Narendra

**Affiliations:** School of Computer Science and Engineering, Vellore Institute of Technology, Chennai, India

**Keywords:** lung nodule segmentation, UNET, residual network, neural network, linear attention mechanism, encoder-decoder

## Abstract

**Introduction:**

Lung cancer remains as an important source of cancer-related mortality worldwide, demonstrating a substantial challenge to public health systems. The absence of evident symptoms in the early stages makes timely diagnosis of lung cancer challenging. Early identification and treatment will reduce the mortality rate caused by lung cancer. Abnormal growths identified as lung or pulmonary nodules can be found in the lungs and some of these could be malignant. A Computer-Aided Detection (CAD) framework can aid in identifying pulmonary nodules by investigating medical images. Automated CAD systems assist radiologists by reducing the diagnostic workload and increasing the possibility of early lung cancer identification. Finding and accurately outlining lung nodules is the specific task of lung nodule segmentation in medical image analysis.

**Methods:**

Multi-scale UNet, Feature Pyramid Network (FPN) with Linear Attention Mechanism and UNet with Asynchronous Convolution Blocks (ACB) and Channel Attention Mechanism were used to segment lung nodules. Multi-scale UNet improvises the traditional UNet architecture by incorporating multi-scale convolutional operations, which improves feature extraction and boosts segmentation accuracy. The UNet with ACB and Channel Attention Mechanism employs a cross-like receptive field that can reduce the impact of redundant information in obtaining representative characteristics. FPN with Linear Attention mechanism uses a multi-scale feature pyramid to identify nodules of different sizes and a linear attention mechanism is employed to improve feature extraction. FPN with Linear Attention mechanism attains a linear time and spatial complexity while effectively segmenting pulmonary nodules.

**Results and discussion:**

Employing the FPN with Linear Attention mechanism yielded the highest performance in the experiments. The highest results in the study using FPN with Linear Attention were achieved using GELU on the LIDC-IDRI dataset with a DSC of 71.59% and IoU of 58.57%. The smooth, probabilistic weighting of GeLU complements the model's attention mechanisms.

## 1 Introduction

**Figure 1:**
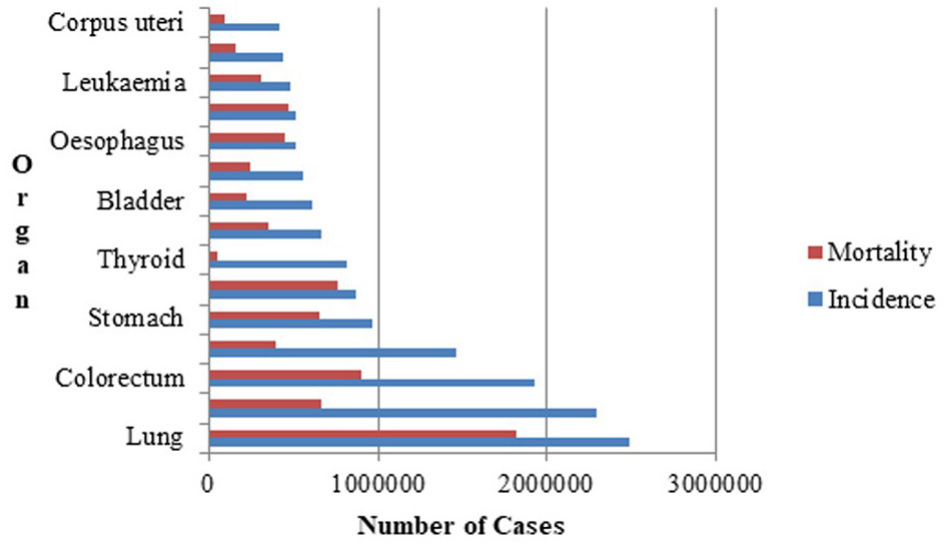
Lung cancer incidence and mortality statistics for both genders in 2022.

The World Health Organization's (WHO) affiliated International Agency for Research on Cancer (IARC) (Ferlay et al., 2024) is devoted to cancer research. According to the latest report in the Global Cancer Observatory (GLOBOCAN) database, which is accessible through the IARC, there were 1,817,469 lung cancer-related deaths and 2,480,675 new cases of lung cancer globally as depicted in Figure 1. The 5-year prevalence is the total number of individuals diagnosed with lung cancer within the last 5 years and still alive at a given time. The 5-year prevalence for lung cancer is is 3,221,461. It is reported that the most common cause of new cancer diagnoses and cancer-related deaths was lung cancer. The estimated number of trachea, bronchus and lung cancer cases worldwide in both males and females, across all age groups from 2022 to 2045 is estimated to be 4.25 million (Ferlay et al., 2024). The significance of lung cancer prevention measures, such as tobacco control and lowering exposure to environmental risk factors, is highlighted by the high incidence rate. Continuous research is required to increase early identification, treatment, and survival rates due to the significant number of new instances of lung cancer.

According to the National Lung Screening Trial (Wu and Raz, 2016), early detection of lung cancer using Computed Tomography (CT) image analysis lowers the death rate from the deadly disease by 20% (Wu et al., 2016). A low-dose CT is recommended for routine screening of high-risk individuals allowing for early detection of potential issues while minimizing radiation exposure. The images are created in real time for medical practitioners to view. To diagnose illnesses, find damage and help with treatment planning, radiologists analyze CT images. Nodules in the lungs are denser than normal tissue on a CT scan (American Thoracic Society, 2025). A CT scan contains a lot of images, so the nodule identification process takes a long time and the radiologists may not agree on the detection of pulmonary nodules. An automated pulmonary nodule identification system resolves this problem by aiding radiologists avoid missing nodules, incorrect diagnoses and also by giving them a second opinion. The appearance, texture, and intensity of pulmonary nodules change depending on their innate qualities (Cao et al., 2020). It is particularly challenging to establish an effective nodule identification approach because of these variations in the pulmonary nodule's appearance and their high degree of similarity to the surrounding tissues.

Lung nodules are frequently early signs of lung cancer, but because they are small (sometimes just a few millimeters) and resemble benign structures, they are difficult to identify. Convolutional neural network's (CNN) ability to recognize patterns helps them spot tiny indicators that human observers or less sophisticated algorithms would overlook (Litjens et al., 2017). Their generalization ability makes them suitable for real-world deployment across a variety of medical imaging contexts, and their hierarchical feature learning guarantees that they can differentiate nodules from false positives (such as calcifications or scarring).

The spatial hierarchies of features can be automatically and adaptively learned from input data by CNNs. In order to identify significant characteristics like edges, textures, and patterns, CNNs employ a mathematical process known as convolution, in which tiny filters or kernels move over the input data. After each convolutional layer, activation functions are applied to introduce non-linearity, helping the network learn complex patterns. Pooling layers are used to lower the computational effort and spatial dimensions while simultaneously strengthening the model's resistance to changes such as minor distortions or shifts.

The proposed work is to explore pulmonary nodule segmentation systems based on multi-scale feature integration architectures such as UNet and FPN by enhancing it for effective segmentation of lung nodules. The contributions in the proposed work to semantically segment lung nodules are as follows:

- Pre-processing techniques using filters like the median and anisotropic diffusion, as well as certain clustering algorithms and morphological processes are used to segment the lungs from the CT scans. The annotations from the dataset are then used to build the nodule masks.
- Multi-scale UNet improvises the traditional UNet architecture by incorporating multi-scale convolutional operations which improves feature extraction and boosts segmentation performance metrics.
- The UNet with ACB and Channel Attention Mechanism employs a cross-like receptive field that can reduce the impact of redundant information in obtaining representative characteristics.
- FPN with Linear Attention mechanism uses a multi-scale feature pyramid to identify nodules of different sizes and a linear attention mechanism to improve feature extraction. FPN with Linear Attention mechanism attains a linear time and spatial complexity while effectively segmenting pulmonary nodules.

The organization of the rest of the paper is as follows. Section 2 discusses the existing work in pulmonary nodule segmentation. The proposed work with different lung nodule segmentation models is described in Section 3. Lung nodule segmentation was performed using a multi-scale UNet, FPN with Linear Attention Mechanism and UNet with ACB and Channel Attention Mechanism. Section 4 discusses the conclusion.

## 2 Related works

Region growing, morphological and energy-based algorithms were traditionally used to identify lung nodules. Region growing techniques make use of region homogeneity and local connectivity, morphological procedures modify pixel structures according to intensity and shape, energy-based techniques control segmentation by minimizing contour energy based on gradients and continuity constraints. In recent years, machine learning and deep learning techniques have been employed to identify nodules. The shape, texture and intensity of nodules are used to extract features that are used for learning in machine learning methods such as Random Forests and Support Vector Machines. By directly learning hierarchical representations from raw image data, deep learning models, in particular CNNs have proven to perform better, enabling end-to-end detection processes.

The basic concept of region-growing algorithms is finding the pixels that most closely match pulmonary nodules and then combining pixels with identical properties. First, a point in each area is identified as the seed point and growth begins from this point. This point is compared to its neighbors, and if they are comparable, they are joined to create a region. The zone expands as a result of this operation until no more pixels are identical. A model that analyzes a region-growing technique based on contrast and fuzzy connection maps (Dehmeshki et al., 2008) is proposed. Nodules with varying contrast levels, nodules close to the lung wall, and nodules connected to blood vessels could all be effectively segmented by the algorithm. The model detected 84% of the nodules, with the remaining nodules being recognized using alternative solutions developed by the authors based on the peripheral contrast vector. A novel toboggan-based automatic segmentation approach (Song et al., 2015) for detecting lung lesions that does not require human interaction or training datasets is proposed. The method has three steps: Automatic Seed Point Selection, Multi-Constraint 3D Lesion Extraction, and Lesion Refinement. This work was tested on the LIDC-IDRI and an in-house clinical dataset and the approach achieved 96.35% detection sensitivity, with an average processing time of less than 8 seconds per lesion. The method is particularly effective for ground-glass opacities, and it outperforms previous algorithms in terms of segmentation accuracy and time efficiency. It shows promise for clinical applications and could be used to segment lesions in other tissues. In future research, the authors suggest to segment lesions in different organs using this approach.

Using a structuring element on the input image to compute or extract the shape or attribute of interest is the basic notion behind morphology-based approaches. A distance map-based technique for segmenting solid nodules connected to blood vessels is introduced by the authors (Diciotti et al., 2011). The authors did not take into account non-solid nodules that were connected to blood vessels. This approach was able to segment 91.7% of the nodules in the LIDC-IDRI dataset and 91% of the nodules from the ITALUNG (Pegna et al., 2009) dataset with some manual refining. An automated erosion strength calculation approach for the morphological opening operation is devised by Kuhnigk et al. (2006), which when combined with the chest wall separation technique allows for the reliable segmentation of tiny, large and irregularly shaped nodules.

The segmentation problem is represented as an optimization problem via energy optimization techniques. The signed distance function-based pulmonary nodule shape model is mapped to the image domain by a set of transformations (Farag et al., 2013). The proposed approach is independent of the type and location of nodules. Even when nodules are connected to other anatomical structures such as arteries or the pleural wall, the alignment and segmentation process is optimized by gradient descent, guaranteeing precise delineation of nodule boundaries. The authors identify Ground Glass Nodules (GGNs) in chest CT images using an asymmetric multi-phase deformable model (Jung et al., 2018). Initially, intensity-based segmentation with histogram modeling extracts the solid and GGO regions, which are refined with a modified energy function and an intensity-constrained averaging function. Multi-scale shape analysis is performed to remove pulmonary vessels. The experimental evaluation using a private dataset and LIDC-IDRI resulted in a DSC of 0.85±0.05 and 0.78±0.07, respectively. The proposed deformable model relies on iterative optimization of level-set functions through energy minimization, which requires repeated computations within the volume of interest. Since it is applied only to small regions and assumes a single nodule per volume, the method is not easily scalable to whole-lung analysis. Moreover, it depends on user input and empirically chosen parameters, introducing variability and limiting automation–for instance, the need for histogram modeling to adjust thresholds. In contrast, the proposed FPN with a linear attention mechanism is trained end-to-end, enabling fully automatic segmentation without case-specific initialization. The model adaptively learns features, reducing reliance on hand-crafted parameters and improving consistency across different CT protocols. Additionally, it effectively handles large inputs, scaling linearly with input size.

In machine learning, features obtained from the input data are used to train a model. Machine learning techniques encompass a wide range of methods that find patterns in data to produce predictions without the need for explicit programming. Support vector machines, fuzzy c-means clustering, and k-means clustering are a few of the frequently employed machine learning approaches. In medical imaging, deep learning has emerged as a potent technique, especially for identifying lung nodules. The segmentation approach by the authors (Hancock and Magnan, 2019) uses machine learning (specifically regression models) to learn the velocity function that guides the segmentation boundary evolution, rather than relying on manually designed functions. Starting from an initial boundary guess, the method iteratively refines the segmentation by estimating the velocity function, which dictates whether the boundary expands (positive velocity inside the nodule) or contracts (negative outside) to align with the true nodule boundary. The initialization process involves local thresholding, connected component analysis, and ray-casting-based radius trimming, calibrated via grid search. The method achieves an average Intersection over Union (IoU) of 0.7185 (±0.1114) and a Dice Score Coefficient (DSC) of 0.8362 (±0.0876) on 112 test nodules of the LIDC-IDRI dataset. However, the approach was sensitive to initialization, computationally slow and not suitable for real-time applications. In order to precisely identify small ground glass opacity pulmonary nodules which are essential for the early diagnosis of lung cancer, the authors (Zhang et al., 2020) introduce a novel image segmentation technique that combines a Bayesian posterior probability difference obtained by a Gaussian mixture model and EM algorithm to improve border detection, with a Markov random field (MRF) energy model to improve contrast and address intensity inhomogeneity utilizing spatial pixel correlations. The method is used inside a level set architecture and verified on clinical and LIDC-IDRI dataset, yielding superior segmentation performance with average IoU scores of 0.7503 and 0.7444, respectively. The findings promote improved medical image analysis by confirming that the suggested approach is reliable and accurate for segmenting small ground glass opacity nodules.

Deep learning models can accurately and automatically identify and categorize lung nodules in chest CT scans by utilizing CNNs. Complex characteristics that are essential for differentiating between benign and malignant nodules, like shape, texture, and location, are learned by these models. By enhancing early lung cancer detection and lowering human error, this technology helps radiologists provide more efficient and timely treatment. A system with two residually connected networks that are incremental and dense respectively with multiple resolutions, enabling automated lung nodule segmentation is proposed by Jiang et al. (2018). They created a multi-scale CNN method for volumetric lung tumor segmentation that allows for precise, automated identification and serial measurement. The proposed work achieved a DSC of 68% on the LIDC-IDRI dataset. A 3D volumetric CNN was designed for processing full CT image volumes which involved 3D convolutions and multi-resolution feature handling across depth, leading to higher memory demands, parameter scaling in practice, and overall resource usage compared to the 2D encoder-decoder architectures discussed in this manuscript. A network with VGG16 and an enhanced Faster R-CNN to identify pulmonary nodules is proposed by Su Y. et al. (2021), and the model obtained an accuracy of 91.2%. Optimization of parameters such as learning rate, batch size, dropout, attenuation coefficient, and step size is performed. The authors (Veronica, 2020) introduce a lung nodule segmentation method using a neural network in conjunction with fuzzy C-means clustering, optimized by a nature-inspired optimization technique and it achieved a sensitivity of 93.3%, specificity of 80%, and accuracy of 86.6% on the ELCAP dataset. The model proposed by El-Regaily et al. (2020) works especially well for locating nodules that are connected to the lung wall or arteries. Without the computational load of full 3D networks, the multi-view CNN efficiently captures 3D information from several 2D perspectives by processing axial, coronal, and sagittal views of each candidate nodule. Lung and nodule segmentation employing a combination of 2D and 3D region growth, thresholding, and morphological procedures is the first step in the detection pipeline. Structures that resemble vessels are removed by examining their unique forms on a three-dimensional depth map. A rule-based classifier is used to filter out obvious non-nodules and a multi-view CNN minimizes false positives. The accuracy achieved by the system was 89.895% on the LIDC-IDRI dataset. The network by Tyagi and Talbar (2022) combined squeeze and excitation modules with a Generative Adversarial Network (GAN) to address class imbalance and the problem of limited data samples while avoiding overfitting. The suggested model achieved a DSC of 80.74% and a sensitivity of 85.46% on the LUNA16 dataset. The proposed network (Bhattacharyya et al., 2023) incorporates a modified UNet architecture based on a bidirectional feature network and the Mish activation function. It combines features from multiple scales and after using the Mish activation function, the DSC increased to 88.89% from 77.84%. Ali et al. (2022) proposed a modified UNet with dense connections and atrous convolution blocks for extracting rich features and the model achieved a DSC of 81% and IoU of 71.6% on the LIDC-IDRI dataset. The features were reusable due to the use of dense connections, and features for various nodule sizes were extracted using a variety of pooling options and transposed convolutions performed at various scales. Ilhan et al. (2023) enhanced lung CT images using a histogram-based non-parametric region localization and enhancement method to highlight abnormal regions and fed the enhanced images into a U-Net model for segmentation of COVID-19-infected lung areas. The model achieved an accuracy, DSC and IoU of 97.75%, 85% and 74% on the COVID19 dataset from Italian Society of Medical and Interventional Radiology. Abiyev and Ismail (2021) presented a methodology employing two CNNs trained via transfer learning, one for binary classification distinguishing pneumonia from normal chest X-ray images, and another for three-class classification identifying COVID-19, pneumonia, and normal cases. The models achieved an accuracy of 98.3%, recall of 97.9%, precision of 98.3% and DSC of 98.0%, indicating effective diagnosis of COVID-19 and pneumonia from chest X-rays from Kaggle repository. This model is desgined for classification rather than segmentation. Zhou et al. (2021) presented a UNet-based approach enhanced with integrated spatial and channel attention mechanisms and focal Tversky loss for segmenting COVID-19 lesions in CT scans. The model achieved a DSC of 83.1% on the dataset from the Italian Society of Medical and Interventional Radiology. The described attention mechanism employs global average pooling for channel weighting and convolutional operations for spatial weighting, enhancing feature selection and segmentation accuracy. However, these pooling and convolution-based methods can be computationally intensive for very high-resolution images. Conversely, linear-attention mechanisms discussed in this manuscript offer better scalability with input size, making them more suitable for processing large-volume CT scans. The proposed work using enhanced UNet, FPN and attention mechanisms discussed in this manuscript provides global context, multiscale feature learning, and computational efficiency. Tang et al. (2025) proposed a transformer based model that uses a partial convolution module in conjunction with a multi-scale attention module. Effective cross-scale feature fusion was accomplished using a channel transformer module and grouping and shuffling concepts were used to enhance feature fusion capabilities. The proposed work obtained a DSC of 91.5% on the LUNA16 dataset and 87.4% on the Tianchi dataset (Tianchi Medical AI Competition:Intelligent Diagnosis of Pulmonary Nodules, 2017). Wang et al. (2022) proposed a two-path model based on enhancing the boundary and a hybrid transformer setup. Global nodule features were obtained, and an edge detection operator was used to generate a boundary enhancement dataset, which improved the boundary precision. The proposed work achieved an average DSC of 89.86% and a sensitivity of 90.50% on the LIDC-IDRI dataset. Liu and He (2024) introduced a parallel fusion model that combined CNNs and Transformer architectures to detect lung nodules in CT scans. This model harnessed CNN's strength in capturing detailed spatial information at high resolution and Transformer's ability to understand global semantic context. It used a pyramid network design with multi-scale features, integrating modules for deep feature interaction and fusion, allowing each architecture to retain its core strengths without adopting the other's framework. The study employed the LUNA16 dataset and achieved a top precision of 95.81% and a robust sensitivity of 93.38% in lung nodule detection. Gautam et al. (2024) present a model incorporating double adaptive attention blocks, which integrate global self-attention from transformers with local spatial details from CNNs. The first block captures global feature statistics, while the second redistributes these features to specific spatial locations, enhancing both global and local context understanding in lung CT images. This design achieved a DSC of 96.57% on the LIDC-IDRI dataset. The approach by Shen et al. (2025) integrates nnU-Net as the core framework with a Transformer decoder, employing a novel data augmentation method that leverages GANs to create synthetic lung nodules. These nodules are dynamically inserted into selected CT regions to enrich training data. The hybrid decoder combines multi-scale feature maps from nnU-Net and progressively refines organ label sets using cross-attention in the Transformer decoder, enhancing segmentation accuracy achieving a DSC of 90.12% on LIDC-IDRI. The model by Shi and Zhang (2025) is an advanced UNet-based model for lung nodule segmentation and it integrates three innovative modules: a Local Aware Attention module that combines deep and shallow features to highlight nodule areas, a Pixel Transformer module that enhances semantic understanding through long-range dependencies, and a Perceptual Adaptation Module that flexibly adjusts feature extraction. This model achieved IoU of 89.6% and DSC of 89.85% on the LIDC-IDRI dataset.

The foundational study by Niknejad Mazandarani et al. (2024) introduced a novel strategy for mitigating noise in low-dose CT (LDCT) imaging, which is essential for accurate lung nodule segmentation. By incorporating a residual multi-scale feature fusion mechanism within a CNN, the work demonstrated how adaptive integration of multi-scale local features and channel dependencies can preserve structural details and high-frequency information, thereby improving image quality for downstream tasks such as nodule detection and segmentation. Building on this foundation, the present work extends the concept of multi-scale feature fusion into specialized architectures for lung nodule segmentation. Multi-scale UNet incorporates multi-scale blocks into the UNet backbone, where convolutional sequences with varying receptive fields capture diverse semantic information, addressing the limitations of fixed receptive fields in conventional UNets. UNet with Asymmetric ACB and Channel Attention further advances residual multi-scale fusion by introducing ACB modules and multi-scale skip connections enhanced with channel attention. This design improves feature representation in high-resolution CT data, eliminating redundant features and effectively fusing multi-scale dependencies, which enhances segmentation precision for small or irregularly shaped nodules. Finally, the FPN with Linear Attention mechanism pushes the methodology forward through an attention aggregation module embedded in the FPN. By adaptively aggregating multi-scale features via attention-guided fusion, this approach alleviates intrinsic shortcomings in feature extraction, achieving fine-grained segmentation of lung nodules in complex CT volumes while maintaining computational efficiency. A comparative summary of the existing lung nodule segmentation methods discussed above is presented in Table 1, highlighting their architectures, datasets, and performance metrics.

**Table 1:** Comparison of methods for lung nodule segmentation.

**References**	**Architecture/method**	**Dataset and metrics**
Jiang et al. (2018)	Multi-scale CNN with residual connections	LIDC-IDRI Average DSC - 68%
Su Y. et al. (2021)	Faster R-CNN model with optimized VGG16	LIDC-IDRI Accuracy—91.2%
Veronica (2020)	Fuzzy C Means with centroid optimization, ANN with OALO	ELCAP, LIDC-IDRI, Marthandam Lung CT Accuracy—86.6%
El-Regaily et al. (2020)	Multiview CNN, Region growing technique	LIDC-IDRI Accuracy—89.89%
Ali et al. (2022)	Dense convolutional blocks, dilated convolutions, UNet	LIDC-IDRI IoU—71.6%
Tyagi and Talbar (2022)	Conditional GAN, Squeeze and Excitation	LUNA16 DSC - 80.74%, Sensitivity—85.46% Local Dataset: DSC—76.36%, Sensitivity—82.56%
Bhattacharyya et al. (2023)	Bi-directional network, UNet, Mish Activation Function	LUNA16 DSC—88.89%
Ali et al. (2022)	Dilated Convolutions at various rates, UNet, Context learning	LIDC-IDRI DSC—81.1%
Suji et al. (2024)	Transfer learning, Pretrained encoders, Encoder-Decoder network	LIDC-IDRI IoU—45%
Tang et al. (2025)	Transformer based model, Grouping and Shuffling concepts	LUNA16 DSC—91.5% Tianchi IoU—87.4%
Wang et al. (2022)	Hybrid CNN-transformer architecture, Down-Attention Sample module	LIDC-IDRI DSC—89.86% Sensitivity—90.50%
Liu and He (2024)	Hybrid CNN-transformer architecture	LUNA16 Precision—95.81% Sensitivity—93.38%
Gautam et al. (2024)	Hybrid CNN-transformer architecture, Adaptive Attention	LIDC-IDRI DSC—96.57%
Shen et al. (2025)	Transformer model, GAN	LIDC-IDRI DSC—90.12%
Shi and Zhang (2025)	Pixel Transformer, Attention	LIDC-IDRI DSC-89.85% IoU—89.6%

Numerous deep learning models have been developed for pulmonary nodule segmentation; however, many existing approaches struggle with generalization across different types of nodules and could benefit from improved efficiency. These limitations highlight the need for a more efficient and comprehensive neural network-based system capable of accurately handling the diverse characteristics of pulmonary nodules. The techniques are computationally intensive, limiting their scalability and real-time clinical deployment especially for resource-constrained environments. While Transformers offer superior accuracy by modeling long-range dependencies, their computational intensity poses challenges, particularly with large images where attention calculations become excessively demanding. In resource-constrained scenarios, CNN-based approaches like FPN paired with a linear attention mechanism as discussed in this manuscript are more advantageous than transformers.

## 3 Proposed methodology of the lung nodule segmentation models

**Figure 2:**
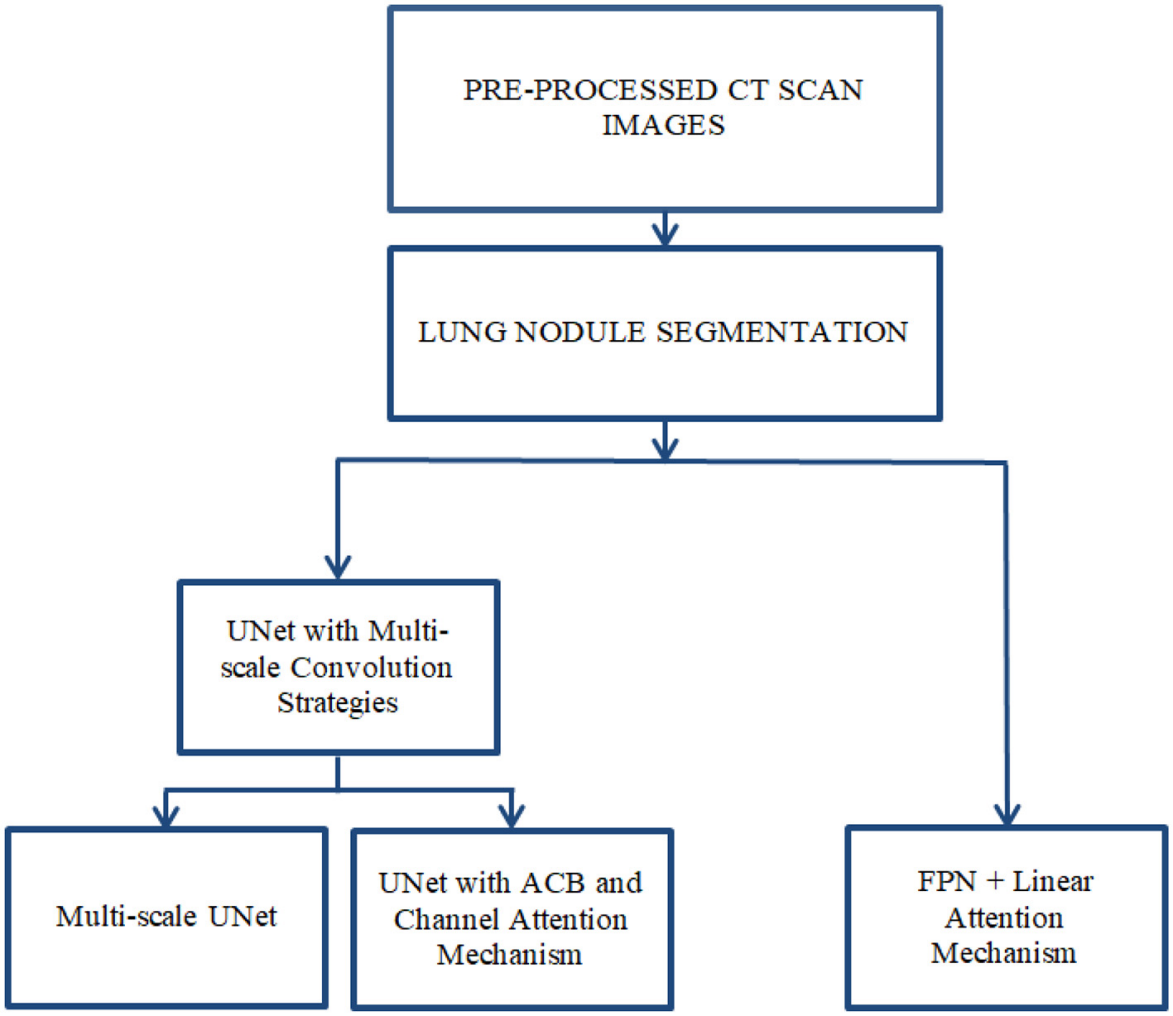
Proposed system design using enhanced UNet and FPN architectures.

This chapter presents the methodology adopted for accurate segmentation of lung nodules from CT scans. The process involves multiple stages, beginning with image pre-processing to enhance image quality and isolate the region of interest (ROI), followed by semantic segmentation using deep learning models as shown in Figure 2. The primary objective is to improve nodule localization accuracy by leveraging advanced CNN architectures, such as multi-scale UNet and FPN, integrated with attention mechanisms.

Lung nodule segmentation refers to the process of outlining and isolating nodules within the lungs from imaging data acquired through any modality, such as CT or X-ray. Semantic segmentation involves labeling each pixel in an image with a class. In the context of lung nodule analysis, this means identifying all pixels that belong to a nodule. Semantic segmentation does not differentiate between individual nodules; it treats all nodules as part of the same class. Lung nodule segmentation was performed using three architectures: Multi-scale UNet, FPN with Linear Attention Mechanism and UNet with ACB and Channel Attention Mechanism. The Multi-scale UNet enhances the conventional UNet framework through the integration of multi-scale convolutional layers, thereby refining feature extraction and elevating segmentation precision. In contrast, the UNet incorporating ACB and Channel Attention Mechanism leverages a cross-shaped receptive field to mitigate the influence of superfluous data, facilitating the capture of more discriminative features. Meanwhile, the FPN augmented with Linear Attention Mechanism exploits a multi-scale feature pyramid to detect nodules across varying dimensions, supplemented by a linear attention module for superior feature refinement. This FPN variant achieves linear computational complexity in both time and space, enabling efficient and accurate segmentation of pulmonary nodules.

### 3.1 Pre-processing

**Figure 3:**
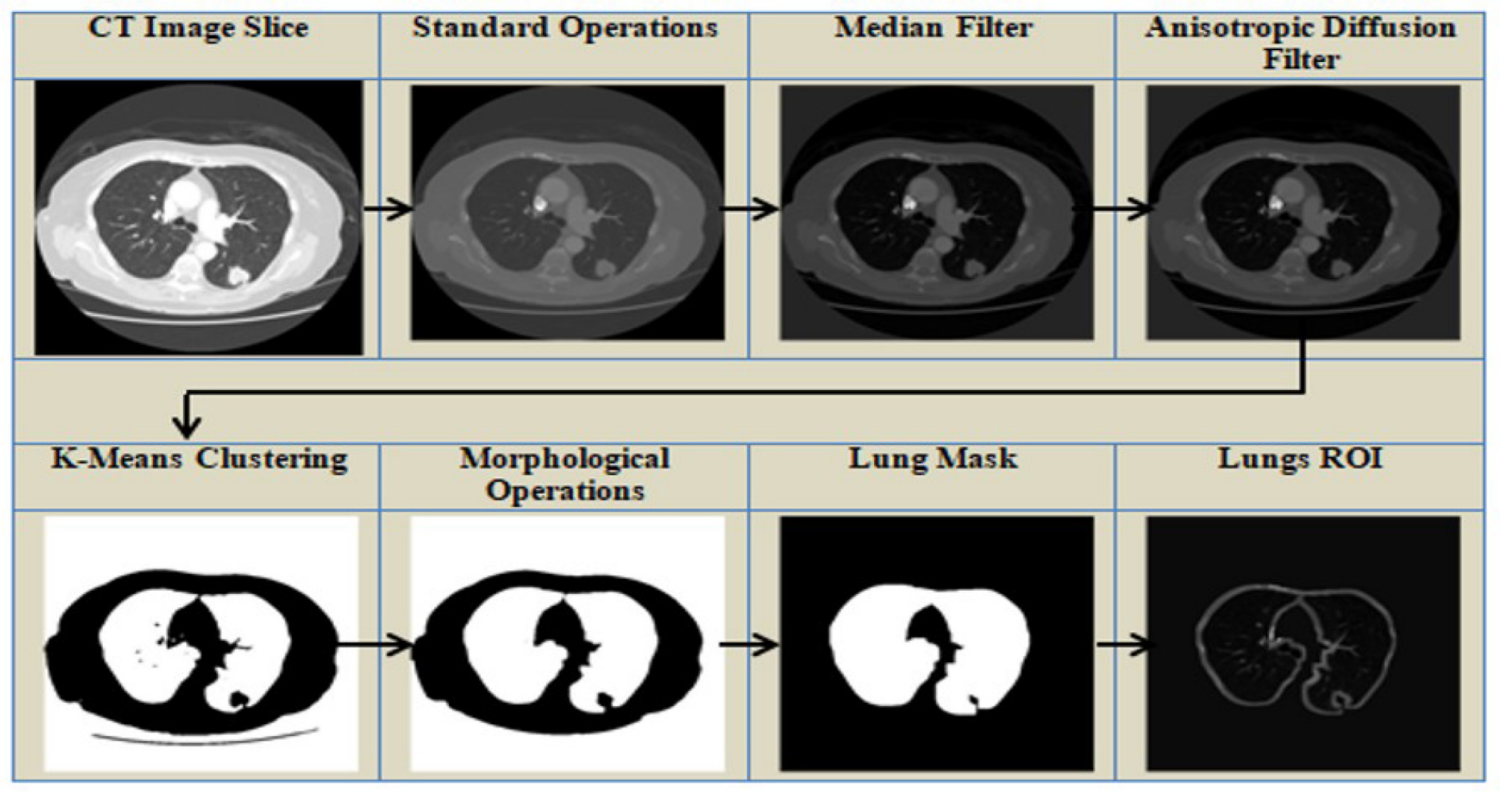
Pre-processing of the CT Scans to segment lungs.

Extraneous objects outside of the ROI are commonly found in medical imaging datasets. Pre-processing of data is typically required to improve its quality and make it more appropriate for further processing. The search space of the model is reduced by extracting a specific ROI, such as lungs from the complete CT scan image. The pre-processing methods used in this study are shown in Figure 3 and are discussed in detail below.

1. **Standardization operation**—To guarantee faster convergence, the original image's average and standard deviation were computed, and the average was subtracted from the image, which was then divided by the standard deviation.
2. **Filtering**—To refine an image and remove noise, the median filter (Pitas and Venetsanopoulos, 1990) and the anisotropic filter (Perona and Malik, 1990) are used. In median filtering, a window is moved across the image, and the window's median values replace the window's center. Median filters minimize noise while preserving edges and are resistant to outliers. This is followed by an anisotropic filter, a technique based on partial differentiation equations that removes noise while preserving edges. The anisotropic diffusion filter makes the homogeneous areas smooth while effectively preserving edges. Anisotropic filters reduce noise, ensure preservation of edges, enable adaptive filtering, and improve structures. The median and anisotropic filters work together to provide a cleaner and more defined image, which leads to improved lung nodule segmentation results.
3. **Clustering**—K-means clustering approach followed by thresholding to separate the lungs from the other entities in the CT scan. Initially, the cluster centroid is chosen at random, and the data points are assigned to the closest cluster. The K-means clustering algorithm is an unsupervised algorithm that classifies data points into groups based on their proximity to the centroid of each cluster, where K denotes the number of clusters. The length of separation between the locations is calculated using the Euclidean distance.
4. **Morphological Processes**—The binary image acquired in the preceding stage is refined with nonlinear morphological procedures such as erosion and dilation. Morphological operations are used to remove distortions from images by utilizing various structuring elements. The structuring element is a matrix that goes over the image and its size determines how many pixels are added or removed. Dilation is a technique for adding pixels to an object's boundary, while erosion is used to reduce the number of pixels on the boundary. Erosion operation is followed by a dilation operation, which is known as opening an image, as illustrated in Equation 1. The symbols θ and ⊕ represent erosion and dilation, respectively. I represents the image, while k is the structural element that moves across it.

(1) $$\begin{array}{l} Iok=\left(I\theta k\right)⊕k \end{array}$$

5. **Segmentation of the lungs and Nodule Mask Generation**—The pixels are designated according to their intensity. If a pixel's value is identical to that of its neighbor, it indicates that they are related, and therefore assigned the same value, and belong to the same region. Bounding boxes are used to define the pixels of a region. The lung mask is extracted using the bounding box's properties. To increase the image size, a dilation operation is used. Finally, multiplying the lung mask by the CT slice results in the lung ROI segmentation. The nodule masks are created using the annotations provided in the dataset.

The pre-processing pipeline enhances segmentation reliability by mitigating noise, intensity variations, and irrelevant background structures in CT images. Intensity normalization standardizes input data, while median and anisotropic diffusion filters reduce scanner noise while preserving key boundaries. K-means clustering, followed by morphological refinements, effectively separates lung parenchyma from adjacent tissues, and region selection eliminates non-lung elements. The resulting lung mask focuses the input on lung fields, minimizing false positives and optimizing model performance on relevant areas. This pre-processing improves tissue boundary contrast and enhances generalization across scans, leading to more precise segmentation.

### 3.2 Lung nodule segmentation methods

After pre-processing, lung nodule segmentation is performed using deep learning architectures based on UNet and FPN frameworks, with enhancements through multi-scale feature extraction and attention mechanisms.

#### 3.2.1 Multi-scale UNet

UNet is a CNN architecture designed primarily for image segmentation, Ronneberger et al. (2015) especially in biomedical and remote sensing applications. It is one of the most widely used models for segmentation tasks. Medical image segmentation has made extensive use of UNet, an encoder-decoder architecture that has achieved notable results. FPN is used to enhance object detection and segmentation, particularly for identifying objects at various scales. It combines high-resolution, spatially rich features from shallow layers with low-resolution, semantically rich features from deep layers to create a multi-scale feature pyramid. The attention mechanism is a concept that enables models to concentrate on the most pertinent aspects of the input data during learning. In this study, segmentation of lung nodules using segmentation models employing enhanced UNet and FPN are analyzed.

**Figure 4:**
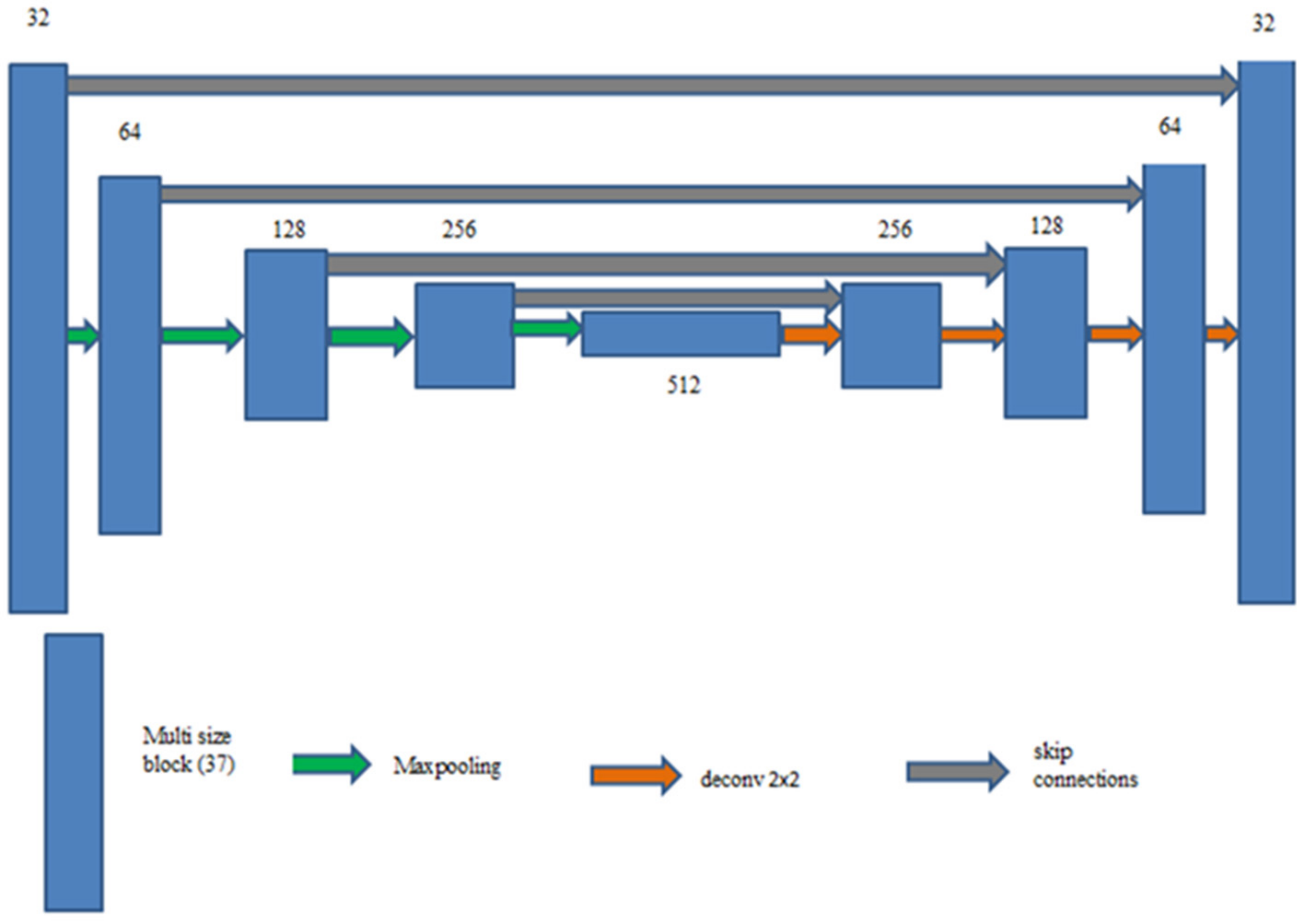
Multi-scale UNet.

Multi-scale UNet improves upon traditional UNet by incorporating multi-scale convolutional operations, allowing better feature extraction. It aims to overcome the limitations of the convolution kernel with a limited receptive field. Semantic characteristics are extracted from the images using a convolution sequence achieving diversity in the features. When the convolution kernel's receptive field is too small, redundant features will be retrieved, whereas when the convolution kernel's receptive field is large, smaller targets are ignored. For instance, in the pulmonary nodule segmentation challenge, the small receptor field makes it difficult to see the structure of the nodule, while the large receptor field makes it difficult to see the edge detail of the smaller nodule. Consequently, it is crucial to process the image using a convolution kernel with various receptive fields. An architecture that combines convolutions of various receptive fields is proposed (Su R. et al., 2021) to obtain good results in the image processing challenge, the network captures a variety of spatial information by integrating multi-scale kernels composed of a 7 × 7 kernel and a 3 × 3 kernel. While the 7 × 7 records more general contextual patterns like structure boundaries, the 3 × 3 kernel retains finer details like edges or textures. The choice of 3 × 3 and 7 × 7 convolutional kernels in our network was guided by the need to balance fine-grained local feature extraction with broader contextual understanding, which is crucial for accurate lung nodule segmentation (Dinh et al., 2023). The 3 × 3 kernels, widely used in CNN architectures such as UNet and ResNet, are efficient at capturing local features like edges, textures, and precise boundaries of small nodules, all while maintaining a low parameter count. In contrast, the 7 × 7 kernels provide a larger receptive field, enabling the network to incorporate wider anatomical context—such as surrounding lung tissue and vascular structures—which is essential for recognizing nodules of various sizes and reducing false positives through contextual awareness. While dilated convolutions can also enlarge the receptive field without increasing the number of parameters, they may introduce gridding artifacts that impair accurate boundary delineation. Our use of explicit 7 × 7 kernels avoids these artifacts, ensuring smoother and more reliable feature representations. Although architectures like Inception blocks combine multiple kernel sizes to capture features at different scales, they come with increased model complexity and computational cost. Similarly, residual multi-scale fusion networks support better gradient flow and feature reuse but require deeper, more complex structures. In contrast, our simplified two-branch design—utilizing both 3 × 3 and 7 × 7 kernels—achieves comparable multi-scale feature integration with significantly lower computational overhead, maintaining a lightweight and efficient architecture. The architecture of the proposed Multi-scale UNet is illustrated in Figure 4. It follows an encoder-decoder design where each encoder block captures contextual features using multi-scale convolutional kernels of 3 × 3 and 7 × 7 sizes. These parallel convolutional paths extract both fine-grained boundary details and broader anatomical context, whose outputs are fused before down-sampling. Skip connections between corresponding encoder and decoder layers facilitate feature reuse, while the decoder progressively upsamples and refines the segmented regions. This multi-scale fusion enables better detection of small and irregular lung nodules compared to the conventional UNet.

#### 3.2.2 UNet with ACB and channel attention mechanism

**Figure 5:**
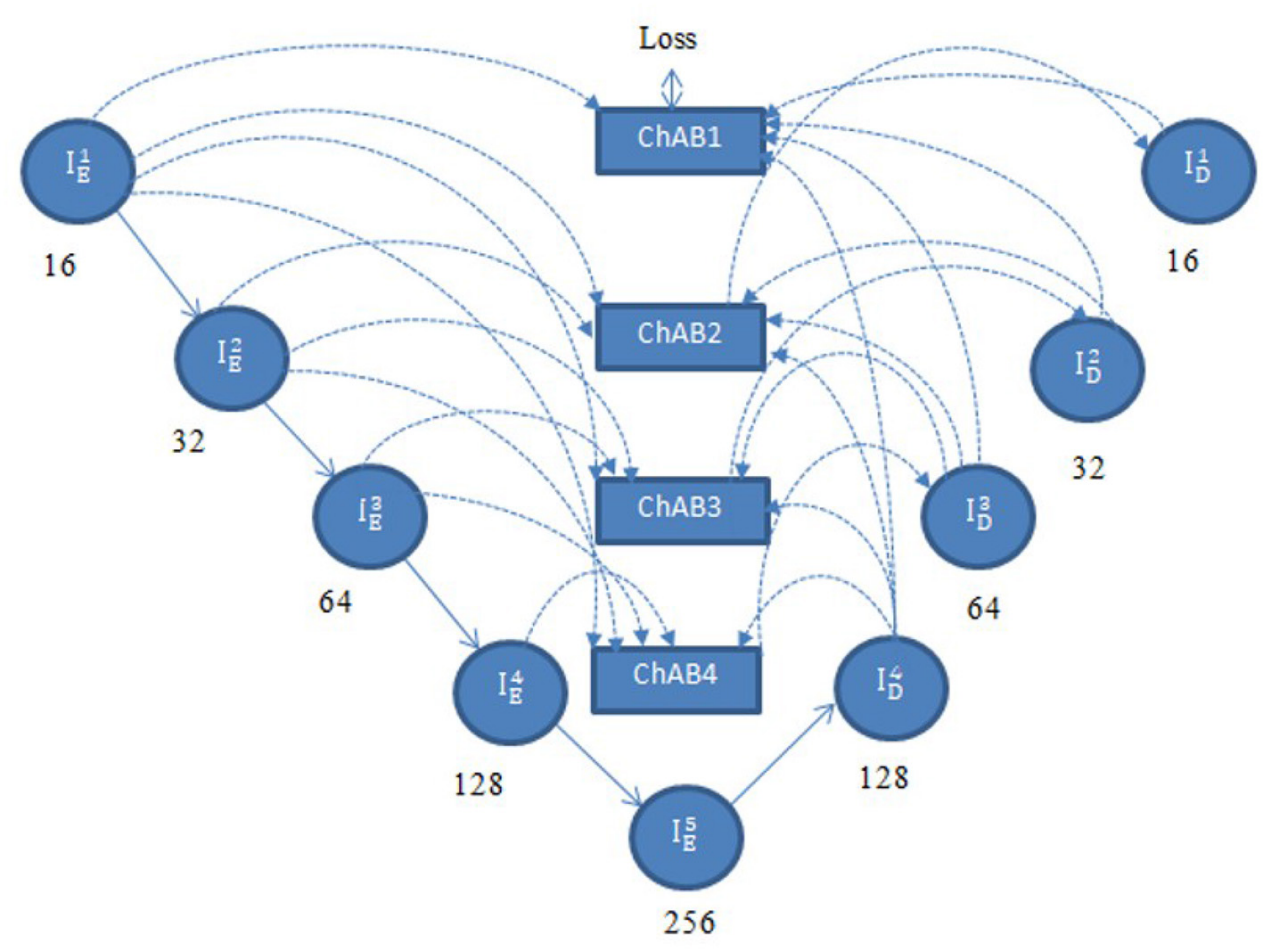
UNet with ACB and channel attention mechanism.

**Figure 6:**
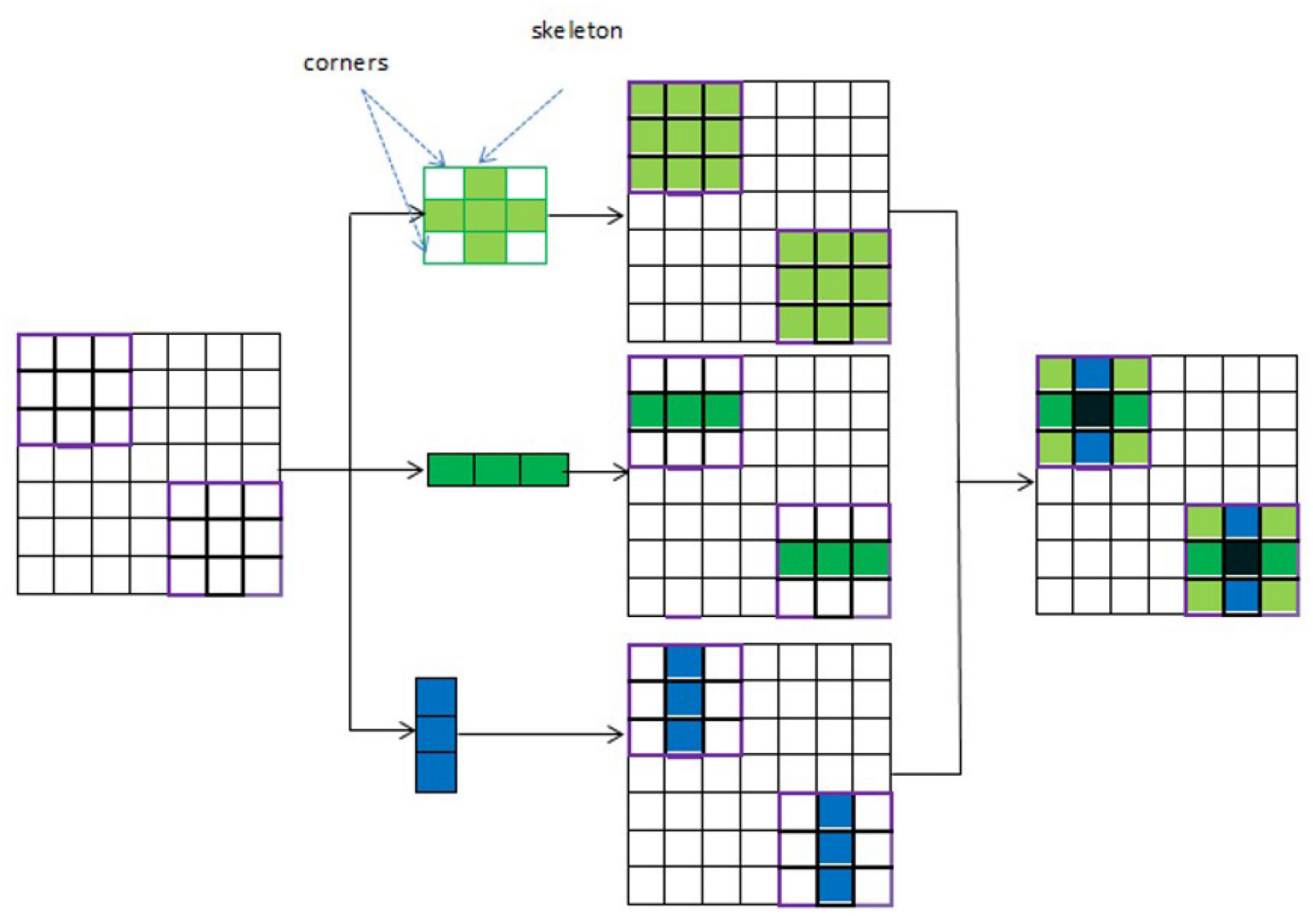
Asynchronous convolution block.

UNet with ACB and Channel Attention Mechanism incorporates a multi-scale skip connected architecture using UNet and an ACB along with channel attention mechanism (Li et al., 2022a), to aggregate the multi-scale features and adaptively realign channel-wise features. The information flow between the encoder and decoder layers are improved and channel attention blocks are employed to adaptively reweigh features from different levels. The multi-scale features were produced by various UNet layers and the semantic features from both low-level and high-level feature maps with various sizes were combined and realigned via the multi-scale skip connections. This helps address the underutilization of features while lowering computational costs. The multi-scale features are integrated by the re-designed skip connections as shown in Figure 5. ACB combines the convolution outputs from the branches of the square, horizontal, and vertical kernels to capture finer details without increasing computing complexity (Ding et al., 2019). Positions on a square convolution kernel's central skeleton are more significant than those on its corners. Therefore, by increasing the weight of the central crisscross sections, asymmetric convolution block improves the representation capability of convolution layers as shown in Figure 6.

#### 3.2.3 ACB

An ACB is a type of CNN module that replaces traditional square convolution filters (e.g., 3 × 3, 5 × 5) with asymmetrical ones, such as 1 × 3 followed by 3 × 1(or vice versa). This technique helps improve model efficiency by reducing computational cost while maintaining performance. To create a cross-like receptive field, three branches are employed: a 3 × 3 convolution, a 1 × 3 convolution (horizontal kernel), and a 3 × 1 convolution (vertical kernel). While the horizontal and vertical kernels ensure the importance of features on the skeleton and increase the network's width, the 3 × 3 convolution identifies features by using a comparatively broad receptive field. The final combined results are obtained by adding the feature maps produced by the three branches. The output is then activated in a nonlinear fashion and the numerical stability is increased by using batch normalization and an activation function. The points in the corners of the kernel provide less information for feature extraction than the weights on the central crisscross places, or the kernel's skeleton, which have larger magnitudes. So, the cross-like receptive field can lessen the impact of redundant information in obtaining representative characteristics. An ACB is created by employing the asymmetric convolutions (Li et al., 2022a) to obtain activation maps from various receptive fields. A cross-like kernel is different from a conventional kernel since it prioritizes the middle crisscross places and de-emphasizes the corners. This results in better management of redundant information, less noise, and more focused feature extraction.

Asymmetric Convolution Block can be formulated as shown in Equations 2 and 3.


(2) $$\bar{i_{j}}=F_{3\times 3}(i_{j}-1)+F_{1\times 3}(i_{j}-1)+F_{3\times 1}(i_{j}-1)$$

(3) $$i_{j}=\sigma \frac{\gamma_{j}(\bar{i_{j}}-Ex(\bar{i_{j}})}{\sqrt{Va(\bar{i_{j}})}+\epsilon_{j}}+\beta_{j}$$

The ACB's input is denoted by *i*_*j*_−1 and its output by *i*_*j*_. The variance function and input expectation are represented by *Va*(·) and *E*_*x*_(·). To provide numerical stability, ϵ is a tiny constant. The normalized result can be scaled by γ and shifted by β, two trainable parameters of the Batch Normalization layer. The activation function is indicated by σ(·). In order to prevent the checkerboard pattern and produce a smooth image, ACB is used to capture and enhance the features in each encoder layer and it is added after each transposed convolution of the decoder. In CNNs, the checkerboard pattern is an artifact that shows up in the output of some architectures, particularly when upsampling is done using deconvolution (transposed convolution) layers. This pattern affects the quality of the output and appears as a grid or checkerboard.

#### 3.2.4 Multi-scale skip connections

**Figure 7:**
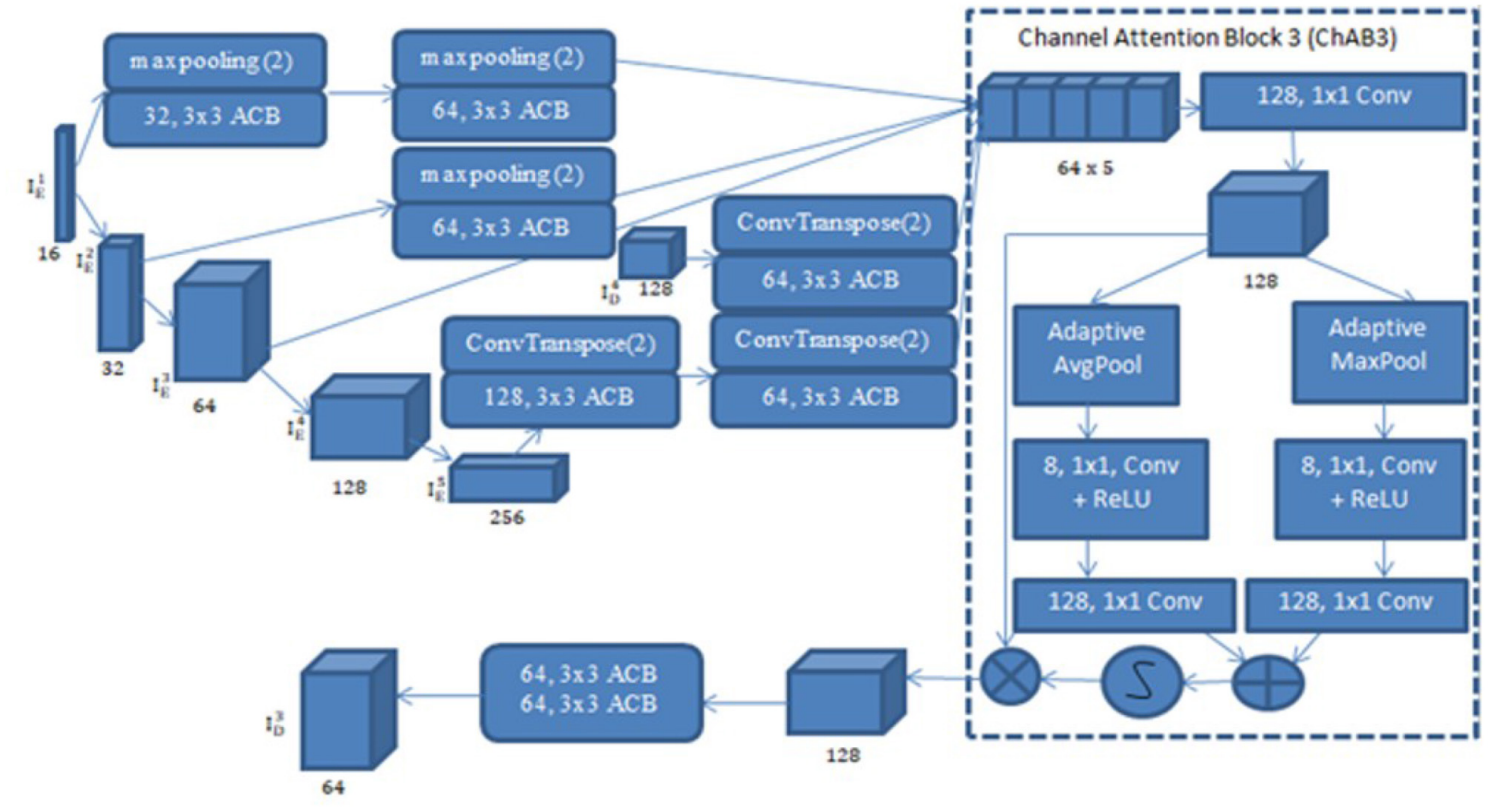
Creation of a multi-scale aggregated activation map $I_{D}^{3}$.

Multi-scale skip connections are employed to capture the interaction between the encoder and decoder, which extracts both fine-grained technical information and coarse-grained semantic information, because the simple connections of UNet do not completely leverage the information at various scales. Figure 7 shows feature maps creation using $I_{D}^{3}$ as an example. First, the feature maps of the same-level encoder layer (i.e., $I_{E}^{3}$) are connected directly, secondly, the fine-grained detailed information contained in lower-level encoder layers (i.e.$I_{D}^{4}$ and $I_{D}^{5}$) are delivered by transposed convolutions and asymmetric convolution blocks. Finally, the coarse-grained semantic information contained in higher-level encoder layers (i.e. $I_{E}^{1}$ and $I_{E}^{3}$) are transmitted by the maxpooling layers and asymmetric convolution blocks. The aforementioned process is expressed in Equation 4.


(4) $$I_{D}^{j}=\{\begin{array}{ll} I_{E}^{j}, & \text{if }j=M\\ \text{ChAB}\left(\left[\underset{\text{Scales: }1^{\text{th}}\sim j^{\text{th}}}{\underset{︸}{\text{Ac}\left(\sum_{k=1}^{j-1}\text{Dg}(I_{E}^{k})\right)}},\underset{\text{Scales: }{\left(j+1\right)}^{\text{th}}\sim M^{\text{th}}}{\underset{︸}{\text{Ac}\left(\sum_{k=j+1}^{M}\text{Ug}(I_{D}^{k})\right)}}\right]\right), & \text{for }j=1,\ldots ,M-1 \end{array}$$

ChAB is the channel attention block which realigns channel-wise features. Asymmetric convolution block is indicated by Ac(·). [·] denotes the concatenation operation, Dg (·) and Ug (·) stand for down-sampling using max-pooling layers and up-sampling using transposed convolution, respectively. ChAB4, ChAB2, and ChAB1 as shown in Figure 7 have a similar connection.

#### 3.2.5 Channel attention block

A Channel attention block (ChAB) is constructed to reweight channel-wise features, and it is based on the Convolutional Block Attention Module (Woo et al., 2018) as shown in Figure 7. The ChAB aims to learn a 1-D weight $W_{ch}\in R^{Ch\times 1\times 1}$ that realigns the channels of the input feature map *F*∈*R*^*Ch*×*H*×*W*^, where Ch, W, and H are the three variables that indicate the channel count, height and width of the activation map. ChAB improves informative channels while limiting indiscriminative ones by multiplying *W*_*ch*_ and F. For $I_{D}^{3}$, we use a 1 × 1 convolution with 128 filters to reduce the number of channels at first. Using $I_{D}^{3}$ as an example, the number of channels are reduced by using a 1 × 1 convolution with 128 filters. The average-pooling and max-pooling processes are then used to compress the spatial dimension. The channels of the squeezed feature maps are reduced to one-sixteenth of their initial size by two convolution layers with eight filters and activation functions. Then, two convolution layers with 128 filters are used to restore the number of channels. Lastly, the sigmoid function activates the sum of the two layers, which is then multiplied by the output of the first convolution. Similarly, the other ChABs yield $I_{D}^{4}$, $I_{D}^{2}$, and $I_{D}^{1}$. The integration of ACB with channel attention was chosen due to their complementary strengths in segmentation tasks. ACB boosts feature representation by emphasizing the central skeleton of convolutional kernels using 3 × 3, 1 × 3, and 3 × 1 branches, enhancing boundary and shape detection without added computational expense. The channel attention block drawing inspiration from CBAM but streamlined, adaptively adjusts channel weights to filter out irrelevant features while effectively merging multi-scale skip connections. Compared to SE, which focuses solely on channel relationships, and CBAM, which adds spatial attention and extra computational load overlapping with multi-scale fusion, our approach optimizes accuracy and efficiency.

#### 3.2.6 FPN and linear attention

**Figure 8:**
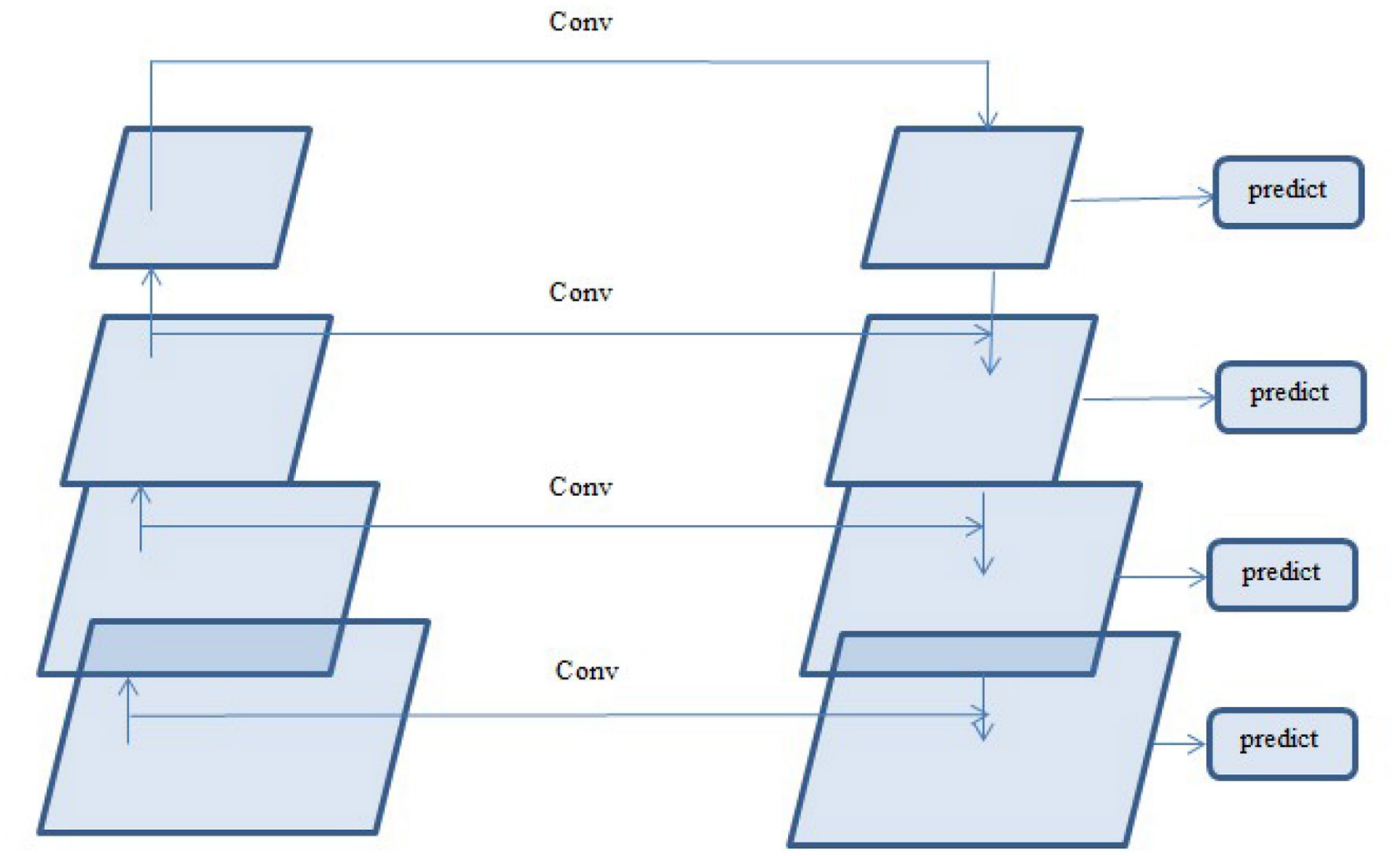
Structure of a feature pyramid network.

**Figure 9:**
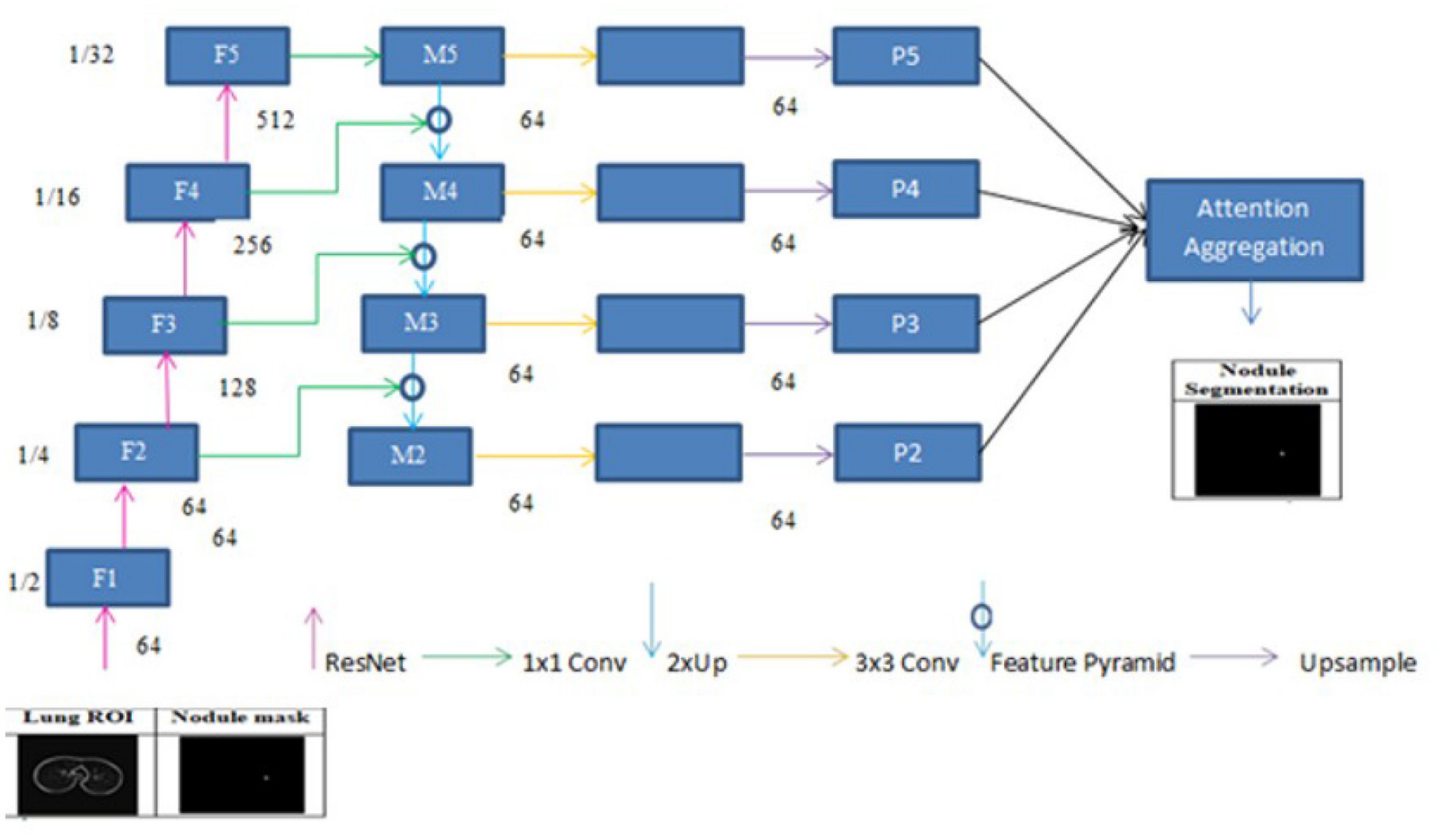
Framework of feature pyramid network with attention aggregation.

A deep learning architecture called FPN is employed in computer vision applications such as segmentation and object detection. By creating a multi-scale feature pyramid, FPN improves feature extraction and makes it possible for a model to recognize objects of various sizes. In order to take advantage of the pyramidal feature hierarchy, the FPN was first created for object detection (Lin et al., 2017). As seen in Figure 8, the FPN's constituent parts are a top-down pathway, a bottom-up pathway, and lateral connections. ResNet is typically used as the backbone of the bottom-up pathway (He et al., 2016), where feature maps created at various scales are used to compute the feature hierarchy. Although they have high-level meanings, the feature maps at the upper pyramid levels are coarse. Up-sampling from high-level feature maps allows the top-down pathway to interpolate fine-resolution features, which are subsequently combined and improved with features of the same spatial scale from the bottom-up pathway through lateral connections. In this study, an architecture that employs FPN enhanced with a linear attention module and an attention aggregation module is used to segment nodules with a linear time and space complexity. As a single end-to-end network, the main constituents of FPN with Linear Attention Mechanism are depicted in Figure 9 as the Attention Aggregation Module, the lateral connections (i.e. the 1 × 1 convolutional layer between the first and second columns), the feature pyramid (i.e. the second and third columns), the bottom-up pathway (i.e. the first column), and the top-down pathway (i.e. the second column) (Li et al., 2022b).

#### 3.2.7 Pathway from the bottom up

ResNet-34 is chosen to create a straightforward and effective framework. The bottom-up pathway creates the feature hierarchy and performs feed forward learning using the ResNet backbone. With a scaling step of two, the feature maps are produced at various spatial resolutions. Large spatial context with coarse resolution is presented at the top levels of feature maps, whereas context information with fine resolution is presented at the bottom levels. The output feature map of each residual block in ResNets is denoted by F2, F3, F4, and F5. The spatial sizes of F2, F3, F4, and F5 are 1/4, 1/8, 1/16, and 1/32 of the input size, respectively. F1 is excluded from the pyramid because of its substantial memory footprint.

#### 3.2.8 Lateral connections and the top-down pathway

**Figure 10:**
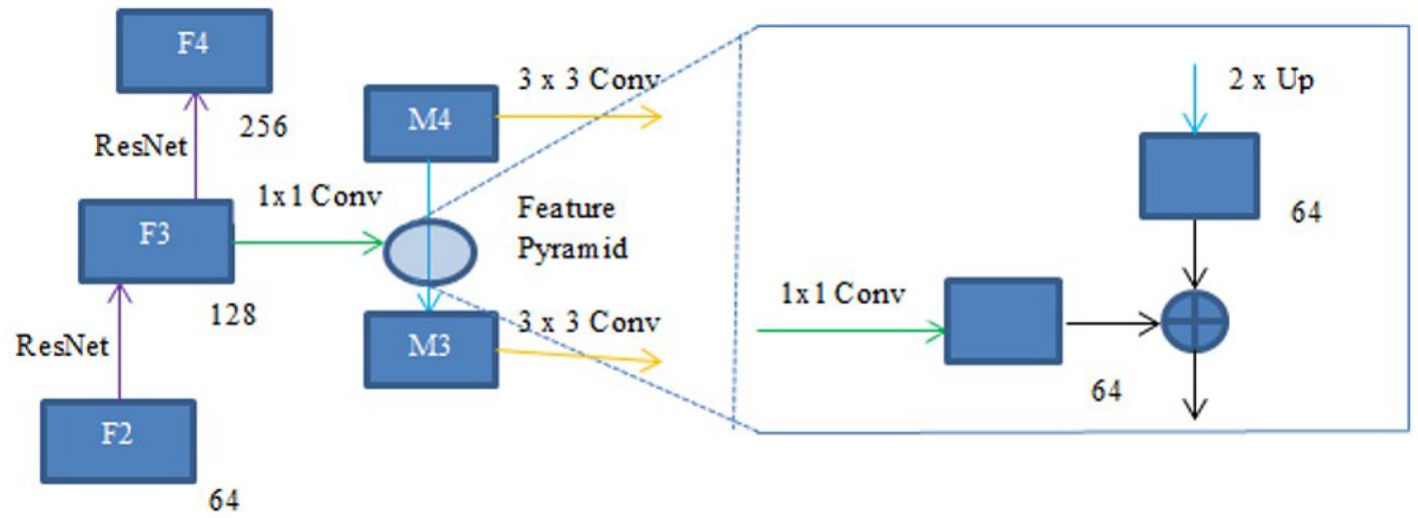
Feature pyramid.

In order to produce fine resolution features, the top-down pathway up-samples semantically rich but spatially coarse feature maps from the top pyramid levels. These characteristics are then combined and enhanced with corresponding features from the bottom-up pathway through lateral connections. A feature pyramid is made up of a top-down layer and a lateral connection, as seen in Figure 10, and M2, M3, M4, and M5 are the feature maps produced. The spatial resolution of a coarse-resolution feature map such as M4 in Figure 10 are upsampled by a factor of 2, with the nearest neighbor up-sampling method. The up-sampled feature map is fused with the corresponding map from the bottom-up pathway through element-wise addition, followed by a 1 × 1 convolutional layer to reduce the channel dimensions. The aforementioned process is repeated until the finest resolution map is produced. To begin the iteration, a 1 × 1 convolutional layer on F5 directly creates the coarsest resolution map (such as M5 in Figure 9). To lessen the aliasing impact brought on by the up-sampling procedure, a 3 × 3 convolution is applied to the merged map created by the associated feature pyramid to create the final feature map. The feature pyramid enhances the representation by including low-level contextual information into spatial feature maps. Since the deep convolution layers have bigger receptive fields than the shallow ones, the high-level features have a large spatial context. Therefore, the low-level features combine with high-level features to learn the multi-scale context information and enhance segmentation accuracy.

#### 3.2.9 Linear attention mechanism

The attention mechanism helps to focus on the significant parts of the input and capture global context. The dot product attention mechanism weighs the input values and generates an output by calculating attention scores that represent the relevance between elements of the input. These scores are then used to calculate a weighted sum of the input values, which forms the output. M represents the length of the input data, calculated as the product of the input data's height (Ht) and width (Wt). The input feature I is represented as $\left[i_{1},\ldots ,i_{M}\right]\in {\mathbb{R}}^{M\times Ch}$. Ch denotes the number of input channels.*D*_*i*_ is the number of channels in the input, *D*_*k*_ is the number of columns in query and key matrices and *D*_*v*_ is the number of columns in the value matrix. The projected matrixes,$W_{q}\in R^{D_{i}\times D_{k}},W_{k}\in R^{D_{i}\times D_{k}}$ and $W_{v}\in R^{D_{i}\times D_{v}}$ are used to produce the query, key and value matrices Q, K and V as shown in Equation 5. The query, key and value vectors are denoted by *q*_*x*_, *k*_*y*_ and *v*_*y*_ respectively.


(5) $$\begin{array}{l} Q=IW_{q}\in R^{M\times D_{k}},K=IW_{k}\in R^{M\times D_{k}},V=IW_{v}\in R^{M\times D_{v}} \end{array}$$

The key matrices and the query must have the same dimensions. Softmax is often employed as the normalization function, and the values are normalized using the normalization function ρ. The similarity between the x-th query feature $q_{x}^{T}\in R^{D_{k}}$ and the y-th key feature $k_{y}\in R^{D_{k}}$ can be represented as $\rho \left(q_{x}^{T}k_{y}\right)\in R^{1}$. Since the key feature and query feature are generated by different layers, the similarity between $\rho \left(q_{x}^{T}k_{y}\right)$ and $\rho \left(q_{y}^{T}k_{x}\right)$ is asymmetric. The dot product attention mechanism is given in Equation 6.


(6) $$\begin{array}{l} Att\left(Q,K,V\right)=\rho \left(QK^{T}\right)V \end{array}$$

ρ is the softmax function used for normalization. Equation 7 illustrates how the x-th row of the resulting matrix based on Equation 6 can be expressed using softmax normalization. Equation 8 displays the first-order approximation of Taylor expansion. Equation 9 illustrates how Equation 7 can be expressed linearly using the first order approximation of Taylor expansion (Li et al., 2020) and L2 norm. *q*_*x*_ and *k*_*y*_ are normalized by L2 norm to ensure that $q_{x}^{T}k_{y}\geq -1$. When the attention is based on the first-order approximation of Taylor expansion, the time and memory complexity becomes linear. To simplify, we replace the softmax function with the first-order approximation of the L2 norm and Taylor expansion (Li et al., 2020).


(7) $$\begin{array}{l} Att{\left(Q,K,V\right)}_{x}=\frac{\overset{M}{\underset{y=1}{\sum }}e^{q_{x}^{T}k_{y}}v_{y}}{\overset{M}{\underset{y=1}{\sum }}e^{q_{x}^{T}k_{y}}} \end{array}$$

(8) $$\begin{array}{l} e^{q_{x}^{T}k_{y}}\approx 1+q_{x}^{T}k_{y} \end{array}$$

(9) $$\begin{array}{l} \text{Att}{\left(Q,K,V\right)}_{x}=\frac{\overset{M}{\underset{y=1}{\sum }}\left(v_{y}+{\left(\frac{q_{x}}{||q_{x}|{|}_{2}}\right)}^{T}\overset{M}{\underset{y=1}{\sum }}\left(\frac{k_{y}}{||k_{y}|{|}_{2}}\right)v_{y}^{T}\right)}{M+{\left(\frac{q_{x}}{||q_{x}|{|}_{2}}\right)}^{T}\overset{M}{\underset{y=1}{\sum }}\left(\frac{k_{y}}{||k_{y}|{|}_{2}}\right)} \end{array}$$

The time and memory complexity utilizing the linear attention mechanism is O(M) since $\overset{M}{\underset{y=1}{\sum }}\left(\frac{k_{y}}{||k_{y}|{|}_{2}}v_{y}^{T}\right)$ and $\overset{M}{\underset{y=1}{\sum }}\left(\frac{k_{y}}{||k_{y}|{|}_{2}}\right)$ from Equation 9 can be computed once and the same can be used for every query. Therefore, the amount of time and space needed would grow linearly with the size of the input image.

#### 3.2.10 Aggregating the attention modules

While global context information is crucial for semantic segmentation, CNN's ability to capture it is severely limited by its local-aware characteristic. The context problem is addressed to an extent using techniques like pyramid pooling. Nevertheless, the contextual dependencies for entire input regions are uniform and non-adaptive, disregarding the differences in local representation of various categories and contextual dependencies. Furthermore, the long-range dependencies of feature maps are not adequately used by those algorithms that are often only used in one layer. FPN is a useful framework for dealing with the problem of multi-scale processing. However, feature maps lack context information due to FPN's designs. Here, a module that combines the attention module is employed to improve long-range interdependence on multi-level in order to collect the global context information as shown in Figure 9 Li et al., (2022b). In particular, the 1 × 1 convolutional layer receives the four feature maps (P2, P3, P4, and P5) produced by the corresponding feature pyramid after they have been concatenated. The global context information is then captured and the fused feature maps are further refined using the linear attention process. Lastly, the original concatenated features are added together with the refined features.

FPN with Linear Attention mechanism integrates a single custom attention mechanism within a module that integrates attention to refine multi-scale features extracted from the encoder-decoder pathway and does not incorporate stacked Transformer encoder-decoder layers. The effective embedding dimension of this attention block corresponds to the concatenated pyramid features, yielding 256 channels, and the mechanism operates in a single-head configuration, where query, key, and value representations are obtained using 1 × 1 convolutions. Thus, the model can be described as a hybrid CNN with single-head attention an embedding size of 256, and 1 attention head without transformer layers. This design choice ensures a lightweight yet effective framework, making it suitable for medical image segmentation tasks where data availability and computational resources are often limited. On CT scans, lung nodules can be small or large and have a variety of shapes. Both fine-grained information (like the borders of tiny nodules) and global context (like bigger nodule structures) must be captured for accurate segmentation. It can be challenging to precisely define boundaries since nodules frequently exhibit low contrast to the surrounding tissues (such as arteries and lung parenchyma) and CT images can be noisy. By integrating semantically rich, low-resolution features (from deeper layers) with high-resolution, low-level features (from shallow layers) through top-down and lateral connections, FPN improves multi-scale feature extraction. Due to its ability to capture both small and larger nodules, this is suitable for lung nodule segmentation. While standard softmax-based self-attention effectively captures long-range dependencies in 2D CT slices, its quadratic time and memory demands render it unsuitable for clinical applications, especially with hundreds of slices per scan. To overcome this, our linear attention module approximates the softmax function, reducing complexity to O(M). This significantly decreases GPU memory usage and speeds up inference. Although its performance is slightly below that of full self-attention or transformer-based segmentation networks, the linear attention approach is better suited for resource-limited environments, enabling faster and more efficient deployment for 2D lung nodule segmentation tasks. The activation function on input i is represented by f(i), In the Gaussian error linear unit (GELU) activation function, the input is weighted according to its probability under a Gaussian distribution as shown in Equations 10 and 11.


(10) $$\begin{array}{l} f\left(i\right)=0.5*i*\left(1+erf\frac{i}{\sqrt{2}}\right) \end{array}$$

(11) $$\begin{array}{l} \mathrm{erf}\left(\frac{i}{\sqrt{2}}\right)\approx \tanh\left(\sqrt{\frac{2}{\pi }}\left(i+0.044715\cdot i^{3}\right)\right) \end{array}$$

Segmenting lung nodules necessitates separating low-contrast nodules from related structures, such as arteries. The minute intensity fluctuations that are essential for precise border delineation are preserved by GeLU's capacity to sustain small negative activations. The FPN with linear attention mechanism architecture avoids problems like dying neurons due to GeLU's smooth, Gaussian-based weighting, which guarantees constant gradient flow. By acting as a soft dropout, GeLU's probabilistic weighting improves generalization and lessens overfitting. The intensity distributions in CT scans, where pixel values frequently follow complex, almost Gaussian patterns as a result of tissue density fluctuations, may be better modeled by GeLU's Gaussian-based methodology. Due to its balance of regularization, smoothness, and sparsity, GeLU has done better than other activations in this study. This makes it a good fit for FPN with a linear attention mechanism in lung nodule segmentation. To enhance semantic segmentation of small or subtle structures, such as lung nodules, and capture long-range dependencies, the FPN with linear attention architecture aggregates the attention blocks and employs linear attention. The time and space complexity increases linearly, linear attention mechanism is significantly faster than dot-product attention and is therefore useful for high-resolution CT slices. It preserves global context awareness, which is essential for comprehending the surrounding tissues.

## 4 Experimental evaluation and performance analysis

### 4.1 Setup for experimentation

Python was used to implement the segmentation models on a system with a GPU and 64 GB of RAM. The models were trained and tested using an NVIDIA GeForce RTX 12 GB GPU. The system was trained for 200 epochs with a batch size of 8 using the Adam optimizer, configured with a learning rate of 1e-5 and a weight decay of 1e-4. Ten percent of the data was used for testing, ten percent for validation, and eighty percent for training. The BCE-Dice Loss function was used and it combined Binary Cross Entropy (BCE) and dice loss Rajput, (2022). The similarity between the model's prediction and the actual segmentation of an image can be calculated using the dice loss. By penalizing incorrect predictions and guaranteeing stability, the Binary Cross Entropy loss function aids in model training by calculating the discrepancy between the actual value and the model's predicted value.

### 4.2 Dataset

The LIDC-IDRI dataset was developed in collaboration with eight medical imaging organizations and seven academic institutes Armato et al., (2011). This publicly available dataset has 1018 DICOM-formatted CT scans. Four radiologists annotate the CT scans in two stages, and the annotations are stored in an XML file. After marking the nodules in the first phase, the radiologists were given access to the other radiologists' anonymized markings in the second phase, after which they provided their final assessment. Because nodules larger than or equal to 3 mm are more likely to be malignant Armato et al., (2011), radiologists label their contours, and these nodules are taken into account in the experiment.

### 4.3 Metrics of performance

Precision quantifies the fraction of correctly predicted positive cases out of all cases the model labeled as positive and is depicted in Equation 12. Recall also known as sensitivity calculates the fraction of actual positive cases that the model accurately identified and is depicted in Equation 13 The segmentation task's performance is gauged by the IoU and DSC. The ground truth (G) and predicted (P) mask images are used to compute DSC and IoU. DSC is equal to two times the area of intersection of G and P divided by the sum of the areas of G and P as shown in Equation 15. IoU is the common area of G and P divided by the combined area of G and P, as shown in Equation 16.


(12) $$\begin{array}{l} Precision=\frac{TP}{TP+FP} \end{array}$$

(13) $$\begin{array}{l} Recall=\frac{TP}{TP+FN} \end{array}$$

(14) $$\begin{array}{l} Accuracy=\frac{TP+TN}{TP+TN+FP+FN} \end{array}$$

(15) $$\begin{array}{l} DSC=\frac{2|G\cap P|}{\left|G|+|P\right|} \end{array}$$

(16) $$\begin{array}{l} IoU=\frac{\left|G\cap P\right|}{\left|G\cup P\right|} \end{array}$$

Precision and recall, while effective for evaluating detection and classification tasks, are less appropriate for medical image segmentation, where the goal is to accurately assess spatial overlap between predicted and ground truth regions. Precision focuses solely on false positives and recall on false negatives, making them sensitive to class imbalance and inadequate for capturing overall segmentation quality. In contrast, the DSC and IoU are preferred in segmentation studies as they directly measure overlap between predicted and reference masks. The DSC, calculated as the harmonic mean of precision and recall, balances false positives and false negatives, while IoU computes the ratio of intersection to the union of the two regions, offering a more stringent evaluation. These metrics are less affected by large background areas common in medical images and provide a single, interpretable score that better reflects segmentation performance. In image segmentation, accuracy as depicted in Equation 14 is not a reliable performance metric due to the significant imbalance between background and object pixels. Since background pixels often dominate an image, a model can achieve high accuracy by predominantly predicting background, even if it misses the objects of interest entirely. This renders accuracy misleading, as it fails to capture the quality of segmentation. Instead, metrics like precision and recall are more effective, as they focus on object pixels–precision evaluates the correctness of predicted regions, while recall assesses how much of the actual region is detected. Overlap-based metrics, such as Dice and IoU, are particularly well-suited for segmentation, as they directly measure the overlap between predicted and ground truth masks, providing a more accurate assessment of performance. If missing nodules poses a greater risk than over-segmenting, recall becomes the most critical metric to prioritize. Emphasizing high recall ensures that the majority of true nodules are identified, thereby reducing the likelihood of overlooking important clinical findings.

### 4.4 Quantitative results and comparisons

A comprehensive quantitative evaluation was conducted to assess the segmentation performance of the three proposed methods: Multi-Scale UNet, UNet with Asynchronous Convolution Blocks (ACB) and Channel Attention Mechanism, and FPN with Linear Attention Mechanism. The evaluation was carried out using the LIDC-IDRI dataset, with the DSC and IoU serving as the primary performance metrics.

The Multi-Scale UNet was first evaluated with various activation functions to determine the optimal configuration. The Leaky ReLU activation yielded the best performance, achieving a DSC of 66.41% and an IoU of 53.92% on the LIDC-IDRI dataset. These results underscore the importance of both network design and choice of activation in improving segmentation outcomes, particularly in handling the heterogeneous appearance and varying sizes of pulmonary nodules. A detailed comparison of activation functions for Multi-Scale UNet is presented in Table 2. Among the tested activations, Leaky ReLU provided superior gradient flow for negative inputs, contributing to improved boundary adherence and overall segmentation accuracy.

**Table 2:** Comparison of precision, recall, DSC and IoU metrics using multi-scale UNet.

**Activation function**	**Precision (%)**	**Recall (%)**	**DSC (%)**	**IoU (%)**
SELU	60.15	65.61	62.71	49.59
ELU	59.49	66.43	62.74	50.24
GELU	60.54	65.96	63.19	50.52
Mish	64.05	67.37	65.64	52.32
Swish	61.48	66.63	63.90	51.59
ReLU	60.78	65.72	63.16	50.72
Leaky ReLU	62.49	71.02	66.41	53.92

Next, the UNet variant incorporating asynchronous convolution and channel attention was evaluated. Here, the ELU (Exponential Linear Unit) activation function provided the highest segmentation accuracy, with a DSC of 65.47% and IoU of 52.56%. This marginal improvement over standard UNet reflects the efficacy of combining skip connections with adaptive channel-wise feature recalibration, which helps address the underutilization of multi-scale features. Table 3 details the results across various activation functions. The improvement with ELU can be attributed to its zero-centered output and smooth non-linear behavior, which enhance learning stability and facilitate better feature integration, especially for images exhibiting diverse or non-linear intensity patterns.

**Table 3:** Comparison of precision, recall, DSC and IoU metrics using UNet with ACB and channel attention mechanism.

**Activation function**	**Precision (%)**	**Recall (%)**	**DSC (%)**	**IoU (%)**
SELU	62.13	66.08	64.03	51.52
ELU	64.42	66.55	65.47	52.56
GELU	59.07	61.37	60.19	48.21
Mish	58.38	61.13	59.79	45.80
Swish	62.74	65.84	64.29	51.39
ReLU	63.28	66.78	64.85	52.01
Leaky ReLU	44.09	53.18	48.20	35.44

The most significant performance gains were observed with the FPN enhanced by a linear attention module. Using the GELU (Gaussian Error Linear Unit) activation function, this method achieved the best results of all tested networks, with a DSC of 71.59% and an IoU of 58.57%. Notably, the probabilistic nature of GELU complements the attention mechanism by enabling smooth and non-binary weighting of features, which is especially beneficial for segmenting structures with complex shapes and subtle contrast differences. Table 4 compares the results for different activation functions, showing consistent improvements with advanced activations like GELU and Mish. The benefit of linear attention lies in its efficient modeling of global context with linear time and space complexity, making it well-suited for processing high-resolution CT slices.

**Table 4:** Comparison of precision, recall, DSC and IoU metrics using FPN with linear attention.

**Activation function**	**Precision (%)**	**Recall (%)**	**DSC (%)**	**IoU (%)**
SELU	66.30	71.85	68.89	55.69
ELU	63.10	77.74	69.22	55.81
GELU	67.50	76.56	71.59	58.57
Mish	64.18	78.92	70.92	57.74
Swish	67.03	72.08	69.60	56.81
ReLU	66.27	72.20	69.96	56.51
Leaky ReLU	67.13	74.56	70.41	57.45

**Figure 11:**
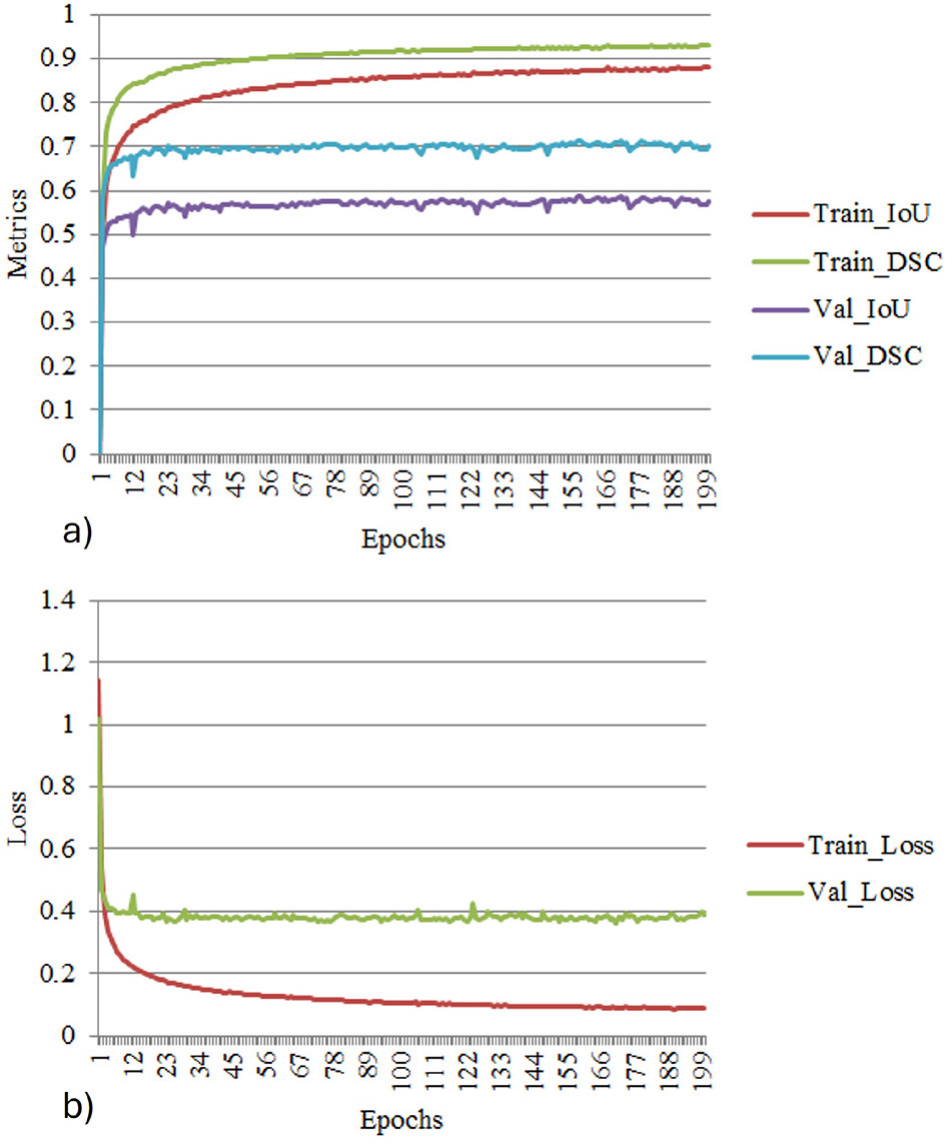
**(a)** Training and validation metrics for FPN with linear attention. **(b)** Training and validation loss for FPN with linear attention.

**Figure 12:**
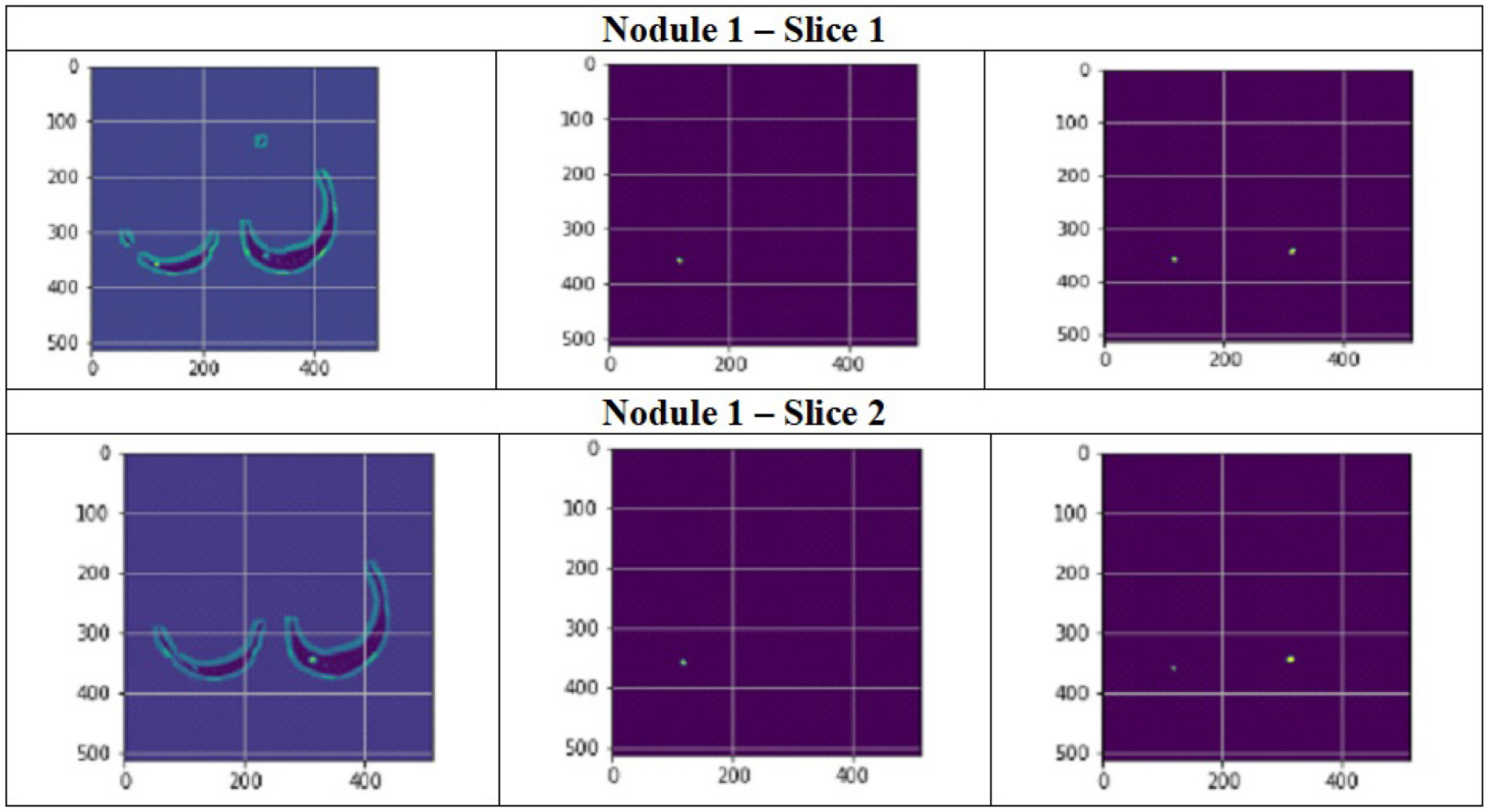
Segmentation results of a patient with nodule 1 using FPN with linear attention (original image, mask image, predicted mask image).

**Figure 13:**
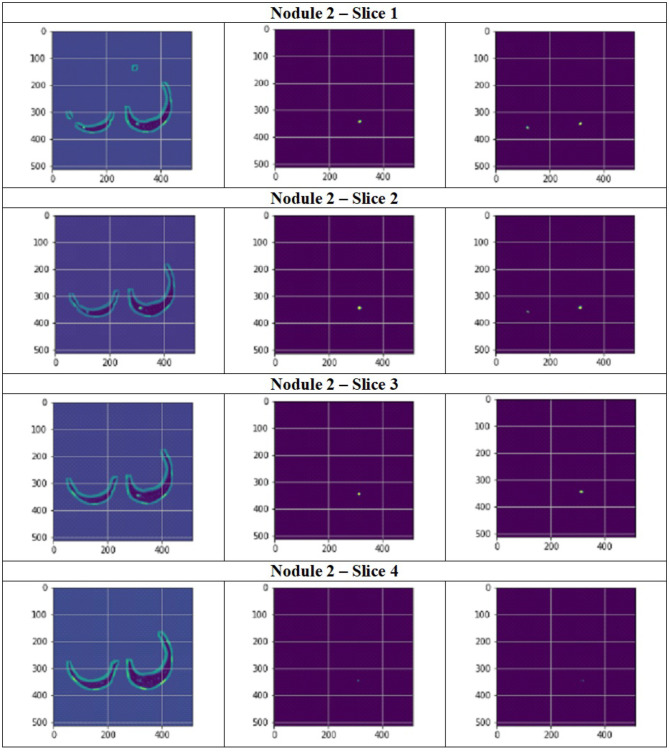
Segmentation results of a patient with nodule 2 using FPN with linear attention (original image, mask image, predicted mask image).

**Figure 14:**
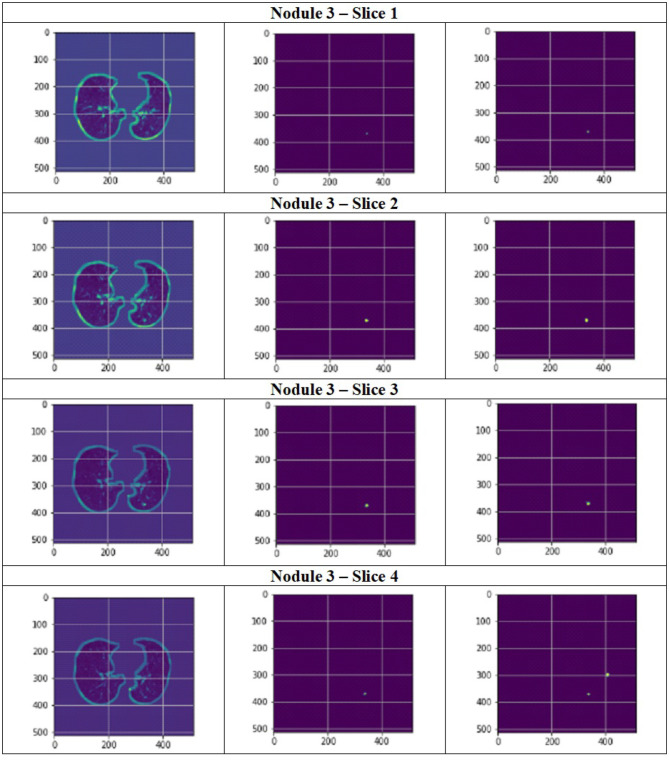
Segmentation results of a patient with nodule 3 using FPN with linear attention (original image, mask image, predicted mask image).

**Figure 15:**
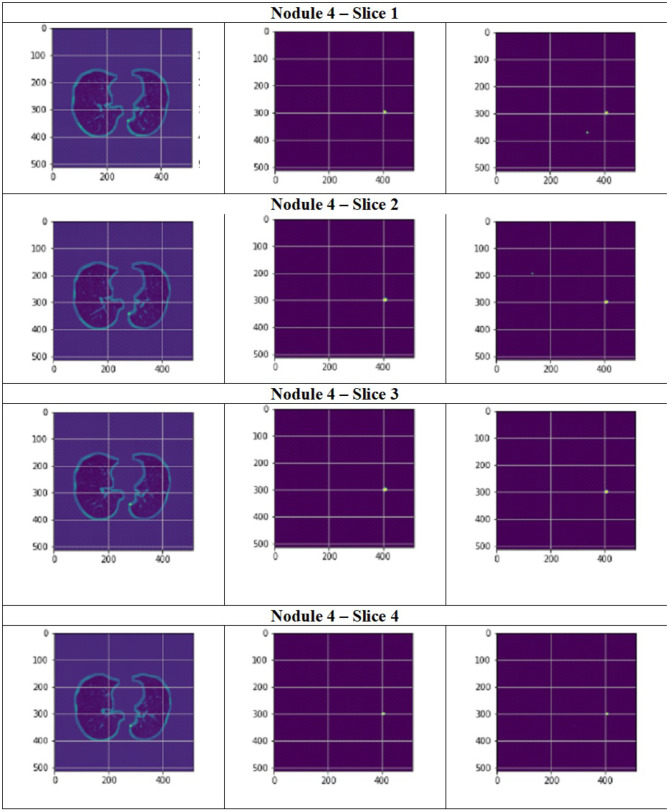
Segmentation results of a patient with nodule 4 using FPN with linear attention (original image, mask image, predicted mask image).

Figure 11 depict the training and validation metrics and loss respectively using FPN with Linear Attention and GELU activation function. The model's training and validation curves indicate convergence, with training IoU and DSC scores plateauing at 0.85 and 0.9, respectively, and validation scores stabilizing at 0.6 and 0.7, showing reasonable generalization to unseen data. Loss curves reveal a sharp initial drop, with validation loss leveling off at 0.35-0.4, while training loss continues to decrease. This suggests the model has learned meaningful patterns, and further training is unlikely to yield significant gains. To contextualize the obtained results, the performance of the proposed models was compared with those reported in prior studies (Tables 5, 6). The proposed FPN with Linear Attention mechanism surpassed the DSC and IoU scores achieved by most existing methods on the LIDC-IDRI dataset, including Mukherjee et al. (2017), Jiang et al. (2018), and Aresta et al. (2019). For example, Mukherjee et al. reported DSCs of 69% for solid nodules and 65% for part-solid nodules, while the highest DSC in this work was 71.59%. This improvement demonstrates that integrating both multi-scale feature extraction and attention-based global context modeling can effectively address persistent challenges in lung nodule segmentation, such as handling nodules that vary in size, shape, and contrast. Visual assessment of the segmentation masks (as shown in Figures 12–15) further highlights the capability of the best performing network to precisely delineate nodule boundaries, even in challenging cases with low contrast or attachment to surrounding tissues. The predicted masks closely match the ground-truth annotations, affirming the model's reliability.

**Table 5:** Comparison of DSC metrics.

**Author**	**DSC (%)**
Mukherjee et al. (2017)	69 ± 14 (solid nodules)
	65 ± 13 (part solid nodules)
Jiang et al. (2018)	68 ± 23
Wang et al. (2022)—Transformer based	89.86
Gautam et al. (2024)—Transformer based	96.57
Shen et al. (2025)—Transformer based	90.12
Shi and Zhang (2025)—Transformer based	89.85
FPN with Linear Attention (2025)	71.59

**Table 6:** Comparison of IoU metrics.

**Author**	**IoU (%)**
Lassen et al. (2015)	52 ± 7 (subsolid nodules)
Aresta et al. (2019)	55 ± 14
Suji et al. (2024)	45
Suji et al. (2021)	45.39
Shi and Zhang (2025)—Transformer based	89.6
FPN with Linear Attention (2025)	58.57

Although standard softmax-based self-attention is effective for modeling long-range dependencies in 2D CT slices, its quadratic time and memory complexity makes it impractical for clinical use, particularly when handling hundreds of slices per scan. To address this, our linear attention module approximates the softmax operation, reducing the complexity to O(M). This substantially lowers GPU memory requirements and accelerates inference. While its performance is somewhat lower than full self-attention or transformer-based segmentation networks, the linear attention design is far more suitable for resource-constrained settings, as the significant computational savings enable faster and more efficient deployment for 2D lung nodule segmentation tasks.

Although Transformers excel in accuracy by capturing long-range dependencies, their high computational demands create challenges, especially for large images where attention computations become resource-intensive. In scenarios with limited resources, CNN-based methods like FPN combined with a linear attention mechanism, as described in the manuscript, provide a more efficient alternative to Transformers.

In summary, the experimental results indicate the following:

- Feature integration at multiple scales, as performed by the Multi-Scale UNet and FPN methods, is crucial for addressing variability in nodule size and appearance.
- Attention mechanisms further enhance model focus, enabling more accurate segmentation, particularly in complex clinical images.
- Advanced activation functions like GELU contribute to stability and generalization, especially when paired with attention modules.
- The proposed FPN with Linear Attention mechanism achieved the highest reported performance on the LIDC-IDRI dataset to date for the evaluated metrics.

Although FPN with linear attention may not match the accuracy of more complex models like full transformers or GANs, its efficiency makes it a practical solution for resource-constrained environments. It facilitates accessible, deployable AI for pulmonary nodule segmentation, potentially boosting early lung cancer detection in areas with limited infrastructure. Future versions could further improve its utility by integrating lightweight quantization methods, such as 8-bit inference. Overall, these findings validate the effectiveness of the proposed methodologies and provide a robust foundation for future improvements and real-world deployment in automated lung nodule analysis.

## 5 Conclusion

Lung nodule segmentation was performed using a multi-scale UNet, UNet with Asynchronous Convolution Blocks and Channel Attention Mechanism and FPN with Linear Attention Mechanism. Multi-scale UNet improves upon the traditional UNet architecture by incorporating multi-scale convolutional operations, which enhance feature extraction and boosts segmentation accuracy. UNet with Asynchronous Convolution Blocks and Channel Attention Mechanism incorporates multi-scale skip connections with adaptive recalibration of channel-wise feature responses by the channel attention mechanisms and employs ACB which comprise square, horizontal, and vertical kernels, enhancing the representational capability of conventional convolutional layers by emphasizing the essential structural components of the receptive field. Despite its success in multi-scale feature representation, the traditional FPN has problems with feature extraction and fusion, especially when it comes to the loss of contextual information during upsampling and merging. So, in FPN with Linear Attention Mechanism, a linear attention system that records global contextual information and enables effective multi-scale feature learning is employed. Through global context modeling, this change enhances the network's ability to extract discriminative representations and makes it possible for the network to encode semantic characteristics across different scales more successfully. The highest DSC and IoU scores was achieved using the FPN with Linear Attention Mechanism in the experiments. It achieved a DSC of 71.59% and IoU of 58.57%. on the LIDC-IDRI dataset using the GELU activation function. Due to its balance of regularization, smoothness, and sparsity, GeLU has done better than other activations in this study. Future work can be done using real time datasets.

## Data Availability

The original contributions presented in the study are included in the article/supplementary material, further inquiries can be directed to the corresponding author.
